# Defining TCRγδ lymphoproliferative disorders by combined immunophenotypic and molecular evaluation

**DOI:** 10.1038/s41467-022-31015-x

**Published:** 2022-06-08

**Authors:** Antonella Teramo, Andrea Binatti, Elena Ciabatti, Gianluca Schiavoni, Giulia Tarrini, Gregorio Barilà, Giulia Calabretto, Cristina Vicenzetto, Vanessa Rebecca Gasparini, Monica Facco, Iacopo Petrini, Roberto Grossi, Nadia Pisanti, Stefania Bortoluzzi, Brunangelo Falini, Enrico Tiacci, Sara Galimberti, Gianpietro Semenzato, Renato Zambello

**Affiliations:** 1 Department of Medicine (DIMED), Hematology and Clinical Immunology Branch, Padova University School of Medicine, Padova, Italy; 2 Veneto Institute of Molecular Medicine (VIMM), Padova, Italy; 3 Department of Molecular Medicine, University of Padova, Padova, Italy; 4 Department of Clinical and Experimental Medicine, Section of Hematology, University of Pisa, Pisa, Italy; 5 Institute of Hematology and Center for Hemato-Oncology Research, University and Hospital of Perugia, Perugia, Italy; 6 Department of Translational Research on New Technologies in Medicine and Surgery, University of Pisa, Pisa, Italy; 7 Department of Computer Science, University of Pisa, Pisa, Italy; 8 CRIBI Biotechnology Center, University of Padova, Padova, Italy

**Keywords:** Gammadelta T cells, T-cell receptor, Chronic lymphocytic leukaemia

## Abstract

Tγδ large granular lymphocyte leukemia (Tγδ LGLL) is a rare lymphoproliferative disease, scantily described in literature. A deep-analysis, in an initial cohort of 9 Tγδ LGLL compared to 23 healthy controls, shows that Tγδ LGLL dominant clonotypes are mainly public and exhibit different V-(D)-J γ/δ usage between patients with symptomatic and indolent Tγδ neoplasm. Moreover, some clonotypes share the same rearranged sequence. Data obtained in an enlarged cohort (n = 36) indicate the importance of a combined evaluation of immunophenotype and *STAT* mutational profile for the correct management of patients with Tγδ cell expansions. In fact, we observe an association between Vδ2/Vγ9 clonality and indolent course, while Vδ2/Vγ9 negativity correlates with symptomatic disease. Moreover, the 7 patients with *STAT3* mutations have neutropenia and a CD56-/Vδ2- phenotype, and the 3 cases with *STAT5B* mutations display an asymptomatic clinical course and CD56/Vδ2 expression. All these data indicate that biological characterization is needed for Tγδ-cell neoplasm definition.

## Abstract (web summary)

Tγδ large granular lymphocyte leukemia (Tγδ LGLL) is a rare lymphoproliferative neoplasm characterized by the expansion of T large granular lymphocytes expressing γδ TCR. Here, based on deep sequencing analysis of the clonotype repertoire, the authors show that leukemic Tγδ cells are characterized by recurrent public clonotypes that are diversified between symptomatic and asymptomatic patients.

## Introduction

In normal conditions, Tγδ lymphocytes represent about 1–10% of peripheral blood CD3+ T-cells. These cells are considered “unconventional” since they are equipped with a T-cell receptor (TCR) composed of a γ- and a δ-glycoprotein chain that, unlike Tαβ cells, does not require the major histocompatibility complex for the antigen presentation. Nevertheless, some authors identified a role of butyrophilin/butyrophilin-like (BTN/BTNL) proteins as specific antigen-presenting molecules to Tγδ lymphocytes^1–3^. According to their Vδ chain rearrangement, two main subsets of Tγδ cells have been identified in humans, i.e., Vδ1 cells and Vδ2 cells, representing more than 95% of Tγδ lymphocytes. In peripheral blood, Tγδ cells are mainly characterized by the Vγ9/Vδ2 rearrangements. In normal conditions, Tγδ lymphocytes lack CD4 and CD8 molecules expression, whereas during cytomegalovirus (CMV) reactivation they can express CD8^4^. Tγδ neoplasms include T-cell large granular lymphocyte leukemia (Tγδ LGLL) and hepatosplenic T-cell lymphoma (HSTCL) whose distinction can be challenging^5,6^.

LGLL is a heterogeneous lymphoproliferative neoplasm characterized by the chronic proliferation of clonal large granular lymphocytes (LGLs) with cytotoxic activity^7,8^. LGLL accounts for 2–5% of chronic lymphoproliferative disorders in Europe and is usually sustained by T-LGLs expressing αβ TCR (Tαβ LGLL), the Tγδ LGLL variant representing only 5% of all T-LGLL. As compared to Tαβ LGLL, the rarest Tγδ LGLL has been less investigated. Single case reports have been published since 1986^9,10^ and few studies on larger cohorts of patients can be found in the literature. Up to now, the largest cohort of Tγδ LGLL patients with 44 cases was reported in 2006 by Sandberg et al.^11^, who observed the preferential expression of the Vγ9/Vδ2 phenotype. Subsequent papers showed that Tγδ LGLL is characterized by a clinical behavior common to Tαβ LGLL^12^ and displays biological similarities with HSTCL^13–15^. Recently, within a cohort of 205 LGLL patients, we studied 23 Tγδ LGLL and demonstrated that the Tγδ variant of the disease shares biological and clinical features with the Tαβ disease subgroup^16^. Moreover, Sanger sequencing obtained in 16 patients of the same cohort showed that similarly to Tαβ LGLL^17–19^ also in Tγδ cases activating mutations in *STAT3* and *STAT5B* genes are present (25% and 19%, respectively) and are mutually exclusive^16^. Besides, other authors recently observed that *STAT3* mutations occur with high frequency in Tγδ LGLL, describing 6 cases all mutated^20^, and 7 mutated cases out of 15^21^.

At variance with Tαβ LGLL, in a large number of Tγδ patients, information is still missing in terms of the underlying TCR clonotypes. In CD8+ Tαβ LGLL^22^ and recently in CD4+ Tαβ LGLL^23^, TCR clonotypes have been reported to be private to the disease (i.e., absent in healthy controls) and to the patients, respectively. The clonotype is identified by the CDR3 region of the TCR chain responsible for antigen recognition, and its analysis is a suitable tool both to investigate the occurrence of a specific peptide/molecule having initially triggered the leukemic proliferation and to unravel its specificity.

In this work, we better characterize Tγδ LGLL patients revealing the public nature of their TCR clonotype pattern as well as a correlation among *STAT3/STAT5B* mutations, the LGL phenotype and the clinical course.

## Results

### Deep sequencing identifies different clonal patterns in Tγδ lymphoproliferations

Deep sequencing analysis of the clonotype repertoire was performed in two HSTCL and nine Tγδ LGLL, including four cases clinically symptomatic and five patients with indolent disease (Table 1). Examination of TCR γ gene rearrangement by IdentiClone TCR assay and TCR next generation sequencing (TCR-NGS) consistently identified at least one major clone in all the 11 cases.

**Table 1:** Results about Tγδ clonal expansions collected by flow cytometry and deep sequencing of TCR gamma and delta chains.

Pts	Disease	% Tγδ cells (on PBMC)	Flow data (Vδ1/Vδ2/Vγ9)	NGS data Vγ-gene	NGS data Jγ-gene	% productive reads	Gamma chain CDR3 Amino Acid Sequence	NGS data Vδ-gene	NGS data Dδ-gene	NGS data Jδ-gene	% productive reads	Delta chain CDR3 Amino Acid Sequence
#1	HSTCL	87%	Vδ1+/Vγ9−	Vγ8	Jγ1/2	97.38%	CATWDSSYYKKLF	Vδ1	Dδ3	Jδ1	99.52%	CALALPGIRGYTDKLIF
#2	HSTCL	95%	Vδ1+/Vγ9−	Vγ3	Jγ1/2	98.20%	CATWDRLHYYKKLF	Vδ1	Dδ2	Jδ1	95.05%	CALGDDIHPLPNTDKLIF
#3	sympt Tγδ LGLL	65%	Vδ1−/Vδ2−	Vγ3	Jγ1/2	83.82%	CATWDRYKKLF	Vδ5	Dδ3	Jδ1	93.23%	CAASAIGSRGTDKLIF
#4	sympt Tγδ LGLL	66%	Vδ1+/Vγ9−	Vγ4	Jγ1/2	61.61%	CATWDGPSMDYYKKLF					
				Vγ8	JγP1	18.89%	CATWDRGGTTGWFKIF	Vδ1	Dδ3	Jδ1	92.98%	CALGEAPLGDTHSDKLIF
				Vγ3	JγP2	5.63%	CATWDRPDWIKTF					
#5	sympt Tγδ LGLL	45%	Vδ1+/Vγ9−	Vγ2	JγP2	40.99%	CATWDGPGSSDWIKTF	Vδ1	Dδ2	Jδ1	51.01%	CALGELVGGPFNTDKLIF
				Vγ2	Jγ1/2	35.66%	CATWDGPSYYKKLF	Vδ1	Dδ3	Jδ1	32.43%	CALGERRGDTFGADKLIF
				Vγ8	Jγ1/2	8.85%	CATWDRWYYKKLF	Vδ1	Dδ3	Jδ1	14.97%	CALGEPPPSLGESKLIF
#6	sympt Tγδ LGLL	26%	Vδ1+/Vγ9−	Vγ2	Jγ1/2	25.11%	CATWDGRVNYYKKLF	Vδ5	Dδ3	Jδ1	17.89%	CAATSSYWGIYTDKLIF
				Vγ2	JγP2	9.18%	CATWDYCSDWIKTF	Vδ1	Dδ2	Jδ1	9.72%	CALGVLPPGVHKLIF
				Vγ4	Jγ1/2	7.12%	CATWEKGKLLYKKLF	Vδ1	Dδ3	Jδ1	9.01%	CALGPFLPTGGYTDKLIF
				Vγ8	JγP2	6.40%	CATWDSSDWIKTF	Vδ1	Dδ2	Jδ1	7.21%	CALGEAAPYQPSYTDKLIF
								Vδ8	Dδ3	Jδ1	6.68%	CAYRSSTLFPYWGIRPDKLIF
#7	ind Tγδ LGLL	58%	Vδ2+/Vγ9+	Vγ9	JγP	32.04%	CALWEVEELGKKIKVF	Vδ2	Dδ3	Jδ1	60.70%	CACDTLLGDTRSNTDKLIF
				Vγ9	Jγ1/2	26.05%	CALERGKLF	Vδ2	Dδ3	Jδ3	38.88%	CACDTLLGDTEDSWDTRQMFF
				Vγ9	JγP	22.93%	CALWEVRELGKKIKVF					
#8	ind Tγδ LGLL	42%	Vδ2+/Vγ9+	Vγ9	JγP	55.65%	CALWEVRELGKKIKVF	Vδ2	Dδ3	Jδ1	98.18%	CACDTVVRGDLNTDKLIF
#9	ind Tγδ LGLL	83%	Vδ2+/Vγ9+	Vγ9	JγP	61.34%	CALWEDRELGKKIKVF	Vδ2	Dδ3	Jδ1	82.50%	CACDTVGLGDTPSTDKLIF
								Vδ2	Dδ3	Jδ1	5.5%	CACDVLGDTTDKLIF
#10	ind Tγδ LGLL	34%	Vδ2+/Vγ9+	Vγ9	JγP	20.75%	CALWEVRELGKKIKVF	Vδ2	Dδ3	Jδ3	39.73%	CACDTSGGHPLSWDTRQMFF
				Vγ9	JγP	19.02%	CALWEEELGKKIKVF	Vδ2	Dδ3	Jδ1	37.66%	CACDTVGLGENGADKLIF
				Vγ9	JγP	12.16%	CALWDTELGKKIKVF	Vδ2	Dδ3	Jδ1	16.96%	CACDSILGALRRSPNTDKLIF
#11	ind Tγδ LGLL	23%	Vδ2+/Vγ9+	Vγ9	JγP	36.53%	CALWEVEELGKKIKVF	Vδ2	Dδ3	Jδ1	39.19%	CACDTVEGWGIQAGDKLIF
								Vδ2	Dδ3	Jδ3	31.07%	CACDSTGEISWDTRQMFF
								Vδ2	Dδ3	Jδ1	7.25%	CACDTLGDTDKLIF
								Vδ2	Dδ3	Jδ3	5.40%	CACDTVRTGGYAWDTRQMFF

*Pts* patients, *TCR* T cell receptor, *PBMC* peripheral blood mononuclear cells, *NGS* next generation sequencing, *HSTCL* hepatosplenic T-cell lymphoma, *sympt* symptomatic, *ind* indolent, *LGLL* large granular lymphocyte leukemia.

NGS increased power allowed a more precise identification of the number of clones included in the T-lymphoproliferation. We observed that four cases had an ultimate monoclonal pattern (both HSTCL cases and two LGLL, i.e., cases #3, #8), whereas in the remaining seven patients NGS revealed a bi or oligoclonal pattern (Table 1).

The main amino acid-productive CDR3 sequences of HSTCL and Tγδ LGLL patients, including immunodominant or codominant and other clones with frequency ≥5% (from now on “major clonotypes”), are reported in Table 1. NGS analysis performed on the two chains of γδ TCR gave similar results on the number and dimension of the major clonotypes in almost all the cases analyzed. The corresponding details on nucleotide composition of the gamma and delta chains are reported in Supplementary Tables 1 and 2, respectively.

### Leukemic Tγδ cells show different Vγ-Jγ and Vδ-Dδ-Jδ usage in symptomatic and asymptomatic patients

**Fig. 1:**
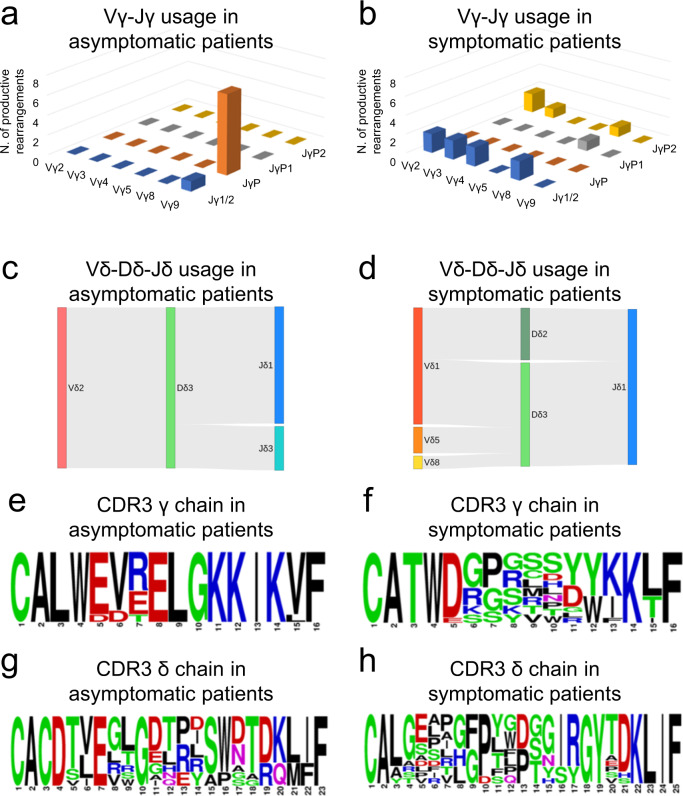
V(D)J rearrangement of gamma and delta chain in asymptomatic (*n* = 5) and symptomatic (*n* = 6) patients affected by Tγδ neoplasm. **a**, **b** Combinatorial usage of VJγ genes (reported in the *x* and *z* axis) shown as histogram of the major productive gamma rearrangements reported in Table 1. **c**, **d** Combinatorial usage of VDJδ genes shown as Sankey plot (vertical bars indicate segments, the connecting stripes the frequency of pairwise combinations) of the major productive delta rearrangements reported in Table 1. Vγ9-JγP (*n* = 8) and Vδ2-Dδ3-Jδ1 (*n* = 8) are the productive combinations most frequently detected among major clones of asymptomatic patients (**a** and **c**), whereas they are absent in symptomatic cases (**b** and **d**). **e**, **h** Gamma and delta CDR3 amino acid composition observed in the asymptomatic (**e** and **g**) and symptomatic group (**f** and **h**) is visualized using WebLogo online tool after sequence alignment performed by ClustalW. Amino acids are colored according to their chemical properties: polar amino acids are shown as green, basic blue, acidic red, and hydrophobic amino acids as black.

Considering the major productive rearrangements among Tγδ LGLL cases, we found that the Vγ9-JγP (*n* = 8/9) and Vδ2-Dδ3-Jδ1 (*n* = 8/12) usage was the combination most frequently found among the major clones, being nearly exclusive in patients characterized by an indolent clinical course (from #7 to #11, Table 1, Supplementary Tables 1 and 2; Fig. 1a and c). On the contrary, in the cases belonging to the symptomatic group (patients #1 to #6), none displayed Vγ9-JγP and Vδ2-Dδ3-Jδ1 usage and a prevalent specific Vγ-Jγ and Vδ-Dδ-Jδ combination was not detected (Fig. 1b and d) whereas the most unconventional genes for peripheral blood TCR repertoire were included. However, a CDR3 similarity in the amino acid composition was recognizable within the symptomatic subset (Fig. 1f and h) as in the asymptomatic group (Fig. 1e and g), the second particularly for gamma chains. Interestingly, considering the whole cohort of patients, the most frequent J gamma gene was Jγ1/2 (41%), followed by JγP (36%), both containing two conserved adjacent lysine motifs (KK) previously reported to be critical for phosphoantigen recognition^24^ (Table 1 and Supplementary Table 1). Analyzing V and J genes among the entire TCR rearrangement repertoire in the group of patients and in a group of 23 healthy controls, no prevalent usage was detected, neither in gamma nor in delta chains (Supplementary Figs. 1 and 2).

### Leukemic Tγδ cells are characterized by recurrent public clonotypes

**Fig. 2:**
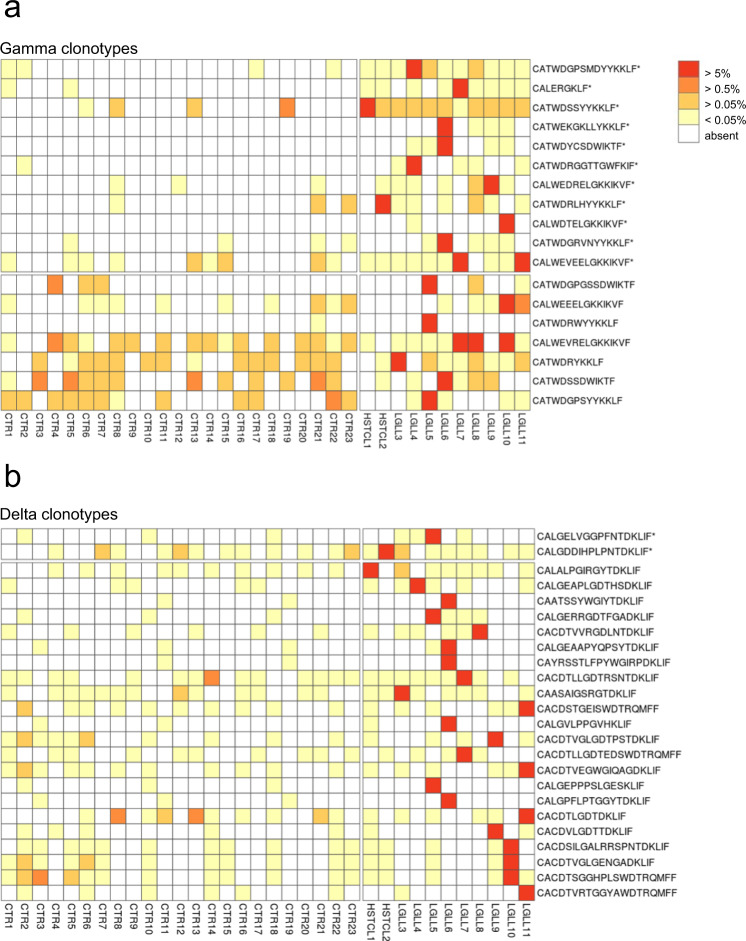
Recurrence of gamma and delta chain major CDR3 sequences of γδ disease patients. Colored squares in the matrices indicate the presence of clonotype sequences in γ (**a**) and δ (**b**) TCR repertoires of the 11 Tγδ neoplasia patients and the 23 healthy controls. The color represents the frequency in the sample. The first 12 gamma clonotypes (**a**) and the first 2 delta clonotypes (**b**) listed are significantly more frequent in patients than in controls (non-parametric Wilcoxon test, one-sided, *P < 0.05). Source data are provided as a Source data file. HSTCL: hepatosplenic T cell lymphoma, LGLL: large granular lymphocyte leukemia, CTR: control.

The patients’ major clonotypes were analyzed for the presence of identical amino acid CDR3 sequences in the gamma and delta chain repertoires of disease cases (*n* = 11) and healthy controls (*n* = 23). We observed that the analyzed clonotypes can be found in both the groups (Fig. 2, detailed data are reported in Supplementary Tables 3 and 4). Only two patients’ gamma clonotypes (CATWEKGKLLYKKLF and CALWDTELGKKIKVF) were private to the disease (i.e., shared only among the patients and not with controls), whereas all the other gamma and delta clonotypes were public (i.e., shared with at least one healthy donor), in contrast to what has been reported for Tαβ LGLL^22,23^. Despite the public nature of patients’ clonotypes, we observed a biased use of CDR3 repertoire, with 12 gamma sequences out of 19 and 2 delta sequences out of 24 being statistically more frequent in patients’ repertoire than in controls (Fig. 2). Consistently, three dominant gamma CDR3 sequences (CATWDSSYYKKLF, CATWDGPSMDYYKKLF, and CALWEVEELGKKIKVF) recurred in the TCR repertoires of all the cases with T neoplasia, both the 9 T-LGLL and the 2 HSTCL patients, whereas in healthy donors were observed in 6 (26%), 4 (17.4%) and 9 (39%) cases, respectively. Of note, one of these sequences belonged to the dominant clone of HSTCL (pt #1) suggesting a similar repertoire among patients with γδ lymphoproliferative disease. Strikingly and in contrast to Tαβ LGLL^22,23^, dominant clonotypes of gamma chain (Fig. 2, red squares) in different patients shared the same rearranged sequence, and this peculiarity was observed within asymptomatic cases: in detail, the CDR3 CALWEVEELGKKIKVF was shared by the dominant clones in patients #7 (32.04%) and #11 (36.53%). Similarly, CALWEVRELGKKIKVF was the dominant or codominant clone in patients #7 (22.93%), #8 (55.65%) and #10 (20.75%) (Table 1). Differently, no recurrence of specific CDR3 sequences was observed among the major delta clonotypes.

Considering the ability of Tγδ cells to recognize bacteria or viruses, we did not identify overlap between the major LGL clonotypes and clonotypes previously linked to the recognition of pathogens, such as M. Tuberculosis^25–27^, and herpes viruses, CMV and Epstein Barr virus^28–31^.

Clustering of the entire TCR repertoire, including major and minor clonotypes, we observed that for γ rearrangements, the patients clustered together separately from healthy controls, whereas for δ rearrangements there was no separation between the two study groups (Supplementary Fig. 3). This result sustains the data collected on only the major clones showing gamma repertoires more similar among patients as compared to healthy subjects.

### Phenotypic and clinical characterization

A detailed immunophenotypic analysis of the Tγδ clone was performed in the enlarged cohort of 39 patients (36 Tγδ LGLL and 3 HSTCL). The flow cytometric evaluation documented an increase of Tγδ cell percentage (16–94% of blood lymphocytes) in all patients (Supplementary Table 5). Among LGLL cases, clone phenotype was distributed in Vδ2+/Vγ9+ (*n* = 21, 58%), Vδ1+/Vγ9− (*n* = 11, 31%) and Vδ1−/Vδ2− (*n* = 4, 11%), with the absolute median number of Tγδ cells equal to 767/µl (interquartile, IQR: 489–1348). Leukemic Tγδ cells expressed the following markers: CD57 (92%), CD16 (92%), CD5 (75%), CD8 (69%), CD56 (53%). As regards the 3 HSTCL, 2 patients were Vδ1+/Vγ9− and 1 Vδ1−/Vδ2−, and Tγδ lymphocytes were CD16+/CD56+/CD57− in all the three cases (Table 2 and Supplementary Table 5). As previously reported^14,32^, CD57 can be considered a marker useful to distinguish Tγδ LGLL from HSTCL.

**Table 2:** Data about the incidence of immunophenotypic, *STAT* mutational, and clinical features in the patients distinguished between HSTCL and Tγδ LGLL (total, symptomatic and asymptomatic patients).

	HSTCL (*n* = 3)	Tγδ LGLL (*n* = 36)			*P* (evaluated between symptomatic and indolent Tγδ LGLL pts)
		All pts (*n* = 36)	Symptomatic (*n* = 17)	Indolent (*n* = 19)	
Characteristics	Number (%)	Number (%)	Number (%)	Number (%)	
CD5+	0/3 (0%)	27/36 (75%)	9/17 (53%)	18/19 (95%)	**0.006**
CD8+	1/3 (33%)	25/36 (69%)	11/17 (65%)	14/19 (74%)	n.s.
HLD-DR	0/3 (0%)	2/36 (6%)	1/17 (6%)	1/19 (5%)	n.s.
CD16+	3/3 (100%)	33/36 (92%)	16/17 (94%)	17/19 (89%)	n.s.
CD56+	3/3 (100%)	19/36 (53%)	4/17 (24%)	15/19 (79%)	**0.002**
CD57+	0/3 (0%)	33/36 (92%)	16/17 (94%)	17/19 (89%)	n.s.
CD158+	3/3 (100%)	14/35 (40%)	7/16 (44%)	7/19 (37%)	n.s.
CD158a+	3/3 (100%)	3/35 (9%)	0/16 (0%)	3/19 (16%)	n.s.
CD158b+	3/3 (100%)	11/35 (31%)	6/16 (38%)	5/19 (26%)	n.s.
CD158e+	2/3 (67%)	4/35 (11%)	2/16 (13%)	2/19 (11%)	n.s.
NKG2+	3/3 (100%)	20/35 (57%)	6/16 (38%)	14/19 (74%)	**0.031**
NKG2A+	0/3 (0%)	17/35 (49%)	3/16 (19%)	14/19 (74%)	**0.002**
NKG2C+	3/3 (100%)	3/35 (9%)	3/16 (19%)	0/19 (0%)	n.s.
Vδ1+/Vγ9−	2/3 (67%)	11/36 (31%)	11/17 (65%)	0/19 (0%)	**<0.0001**
Vδ2+/Vγ9+	0/3 (0%)	21/36 (58%)	2/17 (12%)	19/19 (100%)	**<0.0001**
Vδ1−/Vδ2−	1/3 (33%)	4/36 (11%)	4/17 (24%)	0/19 (0%)	n.s.
CD28	n.d.	0/25 (0%)	0/11 (0%)	0/14 (0%)	n.s.
CD45RA	n.d.	23/25 (92%)	10/11 (91%)	13/14 (93%)	n.s.
CD45RO	n.d.	20/25 (80%)	6/11 (55%)	14/14 (100%)	**0.009**
CD62L	n.d.	6/25 (24%)	0/11 (0%)	6/14 (43%)	**0.020**
*STAT* mutated	3/3 (100%)	10/36 (28%)	7/17 (41%)	3/19 (16%)	n.s.
* STAT3* mutated	0/3 (0%)	7/36 (19%)	7/17 (41%)	0/19 (0%)	**0.002**
* STAT5B* mutated	3/3 (100%)	3/36 (8%)	0/17 (0%)	3/19 (16%)	n.s.
Neutropenia	1/3 (33%)	16/36 (44%)	16/17 (94%)	0/19 (0%)	**<0.0001**
Anemia	2/3 (67%)	9/36 (25%)	9/17 (53%)	0/19 (0%)	**<0.001**
Thrombocytopenia	3/3 (100%)	2/36 (6%)	2/17 (12%)	0/19 (0%)	n.s.
Autoimmune disease	0/3 (0%)	8/36 (22%)	8/17 (47%)	2/19 (11%)	**0.025**
Splenomegaly	3/3 (100%)	4/36 (11%)	4/17 (24%)	0/19 (0%)	n.s.
Secondary neoplasia	0/3 (0%)	8/36 (22%)	5/17 (29%)	3/19 (16%)	n.s.
In therapy	3/3 (100%)	7/36 (19%)	7/17 (41%)	0/19 (0%)	**0.002**
Deceased	2/3 (67%)	1/36 (3%)	1/17 (6%)	0/19 (0%)	n.s.

Statistical *P* values were measured through the χ2 test or Fisher’s test. A value of *P* < 0.05 was accepted as significant. The significant values are reported in bold.

*Pts* patients, *HSTCL* hepatosplenic T-cell lymphoma, *LGLL* large granular lymphocyte leukemia, *n.d.* not detemined, *n.s.* not significant.

The presence of NK receptor KIRs (CD158) characterizing the leukemic clone showed the prevalence of KIR2D2/L3 (CD158b), which was present in 31% of Tγδ LGLL and the 3 HSTCL. As expected^33^, the 3 HSTCL cases were characterized by the expression of multiple KIRs. CD94/NKG2A was expressed in 17 Tγδ LGLL cases (49%), whereas CD94/NKG2C in 3 Tγδ LGLL cases (9%) and all the 3 HSTCL (Table 2 and Supplementary Table 5).

**Fig. 3:**
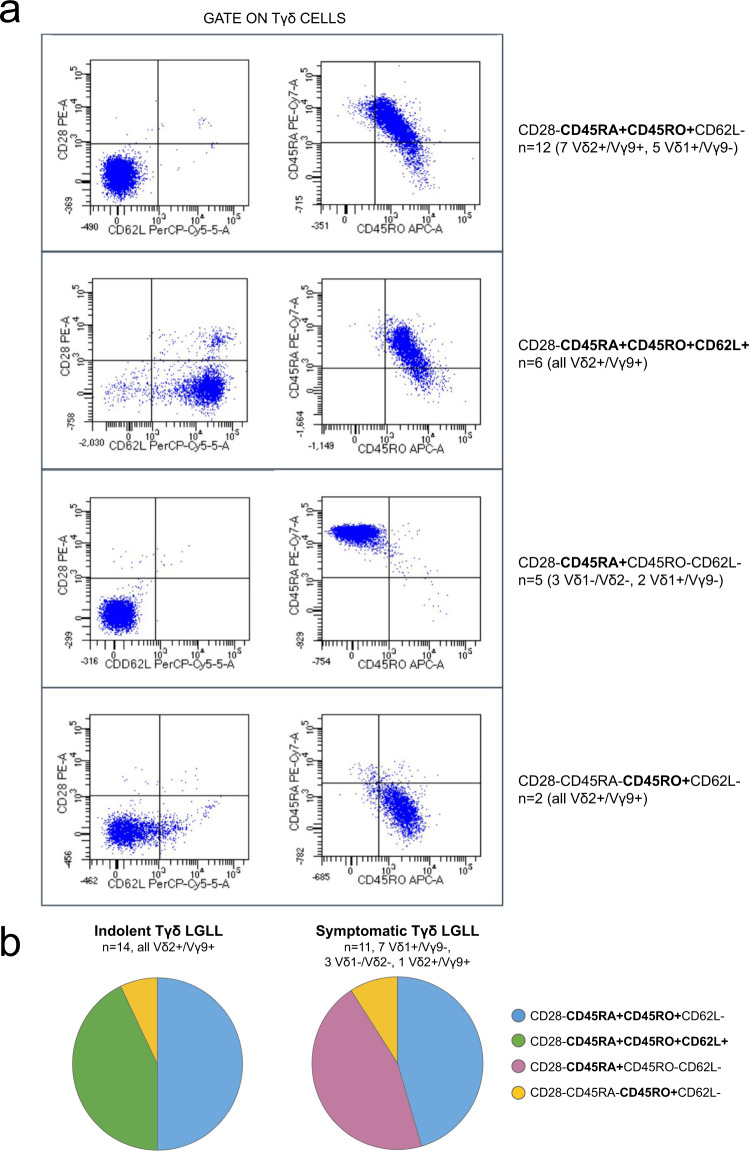
FACS analysis for markers determining the T lymphocyte maturation phase of leukemic clones, CD28, CD45RA, CD45RO and CD62L. **a** Two panels representative for the 4 phenotype combinations found among the Tγδ LGLL cases are reported. **b** The pie charts indicate the distribution of the 4 phenotypes in the patients divided in indolent and symptomatic Tγδ LGLL. LGLL: large granular lymphocyte leukemia.

In the leukemic clones of 25 Tγδ LGLL patients, we also evaluated markers of the lymphocyte maturation (CD28, CD45RA, CD45RO and CD62L; Table 2 and Supplementary Table 5). As expected, since effector/Ag-experienced cells lose CD28 and CD62L, the first marker was absent and the second was found only in 6 cases (24%). On the contrary, CD45RA and CD45RO were frequently co-expressed (in 18 patients, 72%) indicating a prevalence of clone immunophenotype in a transition phase from an effector memory stage toward effector or terminally differentiated effector T lymphocytes. In detail, the phenotypes displaying this co-expression were CD28−CD45RA+CD45RO+CD62L− (*n* = 12) and CD28−CD45RA+CD45RO+CD62L+ (*n* = 6). The remaining phenotypes were: the effector phenotype, CD28−CD45RA+CD45RO−CD62L− (5/25, 20%); and the effector memory CD28−CD45RA−CD45RO+CD62L− (2/25, 8%; Fig. 3). Next, we evaluated whether the double expression CD45RA/CD45RO could be indicative of a transition phase. Time-course data available of 12 patients showed that 8 cases maintained the original phenotype, whereas 4 cases lost CD45RO expression and turned into the effector phenotype CD28−CD45RA+CD45RO−CD62L−.

Among Tγδ LGLL patients, 17 were symptomatic for cytopenia and/or constitutional symptoms (47%) including 7 requiring treatment (19%) during a follow-up ranging between 1 and 11 years (median: 3 years). Symptomatic patients were characterized by neutropenia (94%, *P* < 0.0001), anemia (53%, *P* < 0.001), autoimmune diseases (47%, *P* < 0.05), and splenomegaly (24%, *P* < 0.05). The 3 HSTCL patients were all symptomatic and received specific therapy. The association with autoimmune diseases and secondary neoplasia was observed only in Tγδ LGLL (both in 8/36 patients, 22%; Table 2 and Supplementary Table 6).

The 7 Tγδ LGLL patients requiring treatment received one or more (when refractory to previous therapy) commonly used immunosuppressive drugs, including Cyclosporine A (CyA), Methotrexate (MTX), Cyclophosphamide (CTX), without achieving a complete response in any case. HSTCL patients were treated according to SMILE (steroid, MTX, ifosfamide, L-asparaginase, etoposide) or CHOP (CTX, doxorubicin, vincristine, prednisolone) protocols, without response. One Tγδ LGLL and two HSTCL patients died due to progressive disease during the follow-up (Table 2 and Supplementary Table 6).

### Prevalence of *STAT3* and *STAT5B* somatic mutations in HSTCL and Tγδ LGLL

*STAT3* and *STAT5B* somatic mutation characterization were achieved by Sanger sequencing in all patients and also by targeted NGS in the 11 cases analyzed by TCR-NGS.

All the 3 cases with HSTCL presented *STAT5B* mutations (V712E, N642H, and Y665F, respectively) (Tables 2–3 and Supplementary Table 6), consistent with literature data^34,35^.

**Table 3:** Results about Tγδ clonal expansions collected by flow cytometry and NGS *STAT* mutations screening.

Patients	Disease	% Tγδ cells (on PBMC)	*STAT* mutations^a^	Variant allele frequency %
pt #1	HSTCL	87%	*STAT5B*: V712E	85.99%
pt #2	HSTCL	95%	*STAT5B*: N642H	21.12%
pt #3	symptomatic Tγδ LGLL	65%	*STAT3*: K658R and I659_M660insL	26.79% and 26.35% (monoallelic)
pt #4	symptomatic Tγδ LGLL	66%	*STAT3*: D661Y	41.07%
pt #5	symptomatic Tγδ LGLL	45%	wt	--
pt #6	symptomatic Tγδ LGLL	26%	wt	--
pt #7	indolent Tγδ LGLL	58%	*STAT5B*: N642H and Q706L	17.55% and 14.34%
pt #8	indolent Tγδ LGLL	42%	*STAT5B*: N642H and L643M	10.74% and 10.41% (monoallelic)
pt #9	indolent Tγδ LGLL	83%	*STAT5B*: Y665F	13.03%
pt #10	indolent Tγδ LGLL	34%	wt	--
pt #11	indolent Tγδ LGLL	23%	wt	--

*Pt* patient, *HSTCL* hepatosplenic T-cell lymphoma, *LGLL* large granular lymphocyte leukemia, *PBMC* peripheral blood mononuclear cells, *wt* wild type.

^a^All *STAT3* and *STAT5B* mutations were confirmed by Sanger sequencing, the peaks of the mutated nucleotides were consistent with the VAF measured by NGS.

Among Tγδ LGLL, 10/36 cases (28%) carried *STAT3* or *STAT5B* mutations in a mutually exclusive way (Table 2). In detail, 7 patients had *STAT3* mutations: K568R and I659_M660insL (pt #3), 3 D661Y (pts #4, #33 and #37) and 3 Y640F (pts #29, #35 and #36); and 3 patients had *STAT5B* mutations: N642H and Q706L (pt #7), N642H and L643M (pt #8) and Y665F (pt #9) (Table 3 and Supplementary Table 6).

*STAT* mutational data obtained by NGS on peripheral blood mononuclear cells (PBMC) has been related to the frequency of Tγδ population determined by flow cytometry on PBMC samples and to the clones defined by TCR-NGS (Table 1). We observed that in HSTCL case #1, showing Tγδ expansion in PBMC of 87% according to flow cytometry and only one clone of 95% according to TCR-NGS, the variant allele frequency (VAF) of the *STAT5B* mutation (86%) suggests its clonal presence in a homozygous or hemizygous fashion. In case #3 the size of the T-cell clone was consistent with *STAT3* mutations being heterozygously present in all or most cells of the leukemic population. In contrast, in patients #2, #8, and #9, *STAT3* and *STAT5B* mutations appeared subclonal, present in a smaller fraction of the leukemic population. In cases #4 and #7, who are characterized by oligoclonality, the clonal location and hetero or homozygous presence of the mutation cannot be established.

### Correlation between clinical and biological features

We observed that in Tγδ LGLL patients presenting an indolent behavior (*n* = 19), as compared to those symptomatic (*n* = 17), leukemic clone was more frequently characterized by CD5 (95% vs 53%, *P* < 0.01) and CD56 (79% vs 24%, *P* < 0.01) positivity, NKG2A expression (74% vs 19%, *P* < 0.01) and Vδ2/Vγ9 rearrangement (100% vs 12%, *P* < 0.0001; Table 2 and Supplementary Table 5). On the contrary, the absence of Vδ2/Vγ9 expression, including both Vδ1+ and Vδ1−/Vδ2− cases, correlated with more severe Tγδ LGLL diseases (15/17, 88%) as compared to asymptomatic patients (0/19, 0%, Table 2 and Supplementary Table 5). Similarly, in all the 3 HSTCL patients leukemic Tγδ cells lacked Vδ2 rearrangement, 2 being Vδ1+ and the remaining Vδ1−/Vδ2−. Moreover, NKG2C was detected on Tγδ cells only among symptomatic Tγδ LGLL patients (19%) and in all the 3 HSTCL (Table 2 and Supplementary Table 5). The absolute median number of Tγδ cells was 1133/µl (IQR: 424–2696) in symptomatic patients and 724/µl (IQR: 518–944) in cases with indolent disease (Supplementary Table 6).

Out of 25 Tγδ LGLL patients evaluated for lymphocyte maturation markers, 11 were symptomatic and 14 asymptomatic. Of note, the CD28−CD45RA+CD45RO+CD62L+ (*n* = 6) phenotype was found only in association with asymptomatic patients with leukemic cells expressing Vδ2/Vγ9 (*P* < 0.05), whereas the effector phenotype CD28−CD45RA+CD45RO−CD62L− (*n* = 5) was found exclusively in the symptomatic group and is associated to Vδ2/Vγ9 negativity (*P* < 0.01; Fig. 3b; Supplementary Table 5).

All the 7 Tγδ LGLL patients with *STAT3* mutations were characterized by CD56− LGLs (*P* < 0.01, Supplementary Table 5) and symptomatic disease (100%, *P* < 0.05), mainly neutropenia (Supplementary Table 6). Indeed, in Tγδ LGLL there was a very significant association between *STAT3* mutations and neutropenia, as the latter was observed much less frequently in *STAT3* wild-type cases (8/29, 28%; *P* < 0.001, Supplementary Table 6). In contrast, all the 3 Tγδ LGLL patients with *STAT5B* mutations are included in the group presenting CD56+, analogous to the CD56 expression observed in *STAT5B* mutated HSTCL patients^35^, and NKG2A+ phenotype and asymptomatic disease (*P* = n.s., the small number of mutated cases prevents a proper statistical assessment). Interestingly, *STAT5B* mutations in 2 Tγδ LGLL cases (pts #8 and #9) displayed low VAF compared to their clones suggesting a later onset of these mutations in clone genesis (Tables 1 and 3). Since *STAT5B* mutations were associated with indolent behavior in Tγδ LGLL and with aggressiveness in the 3 HSTCLs (Table 2 and Supplementary Table 6), the association of *STAT5B* to a clinical course cannot be established.

## Discussion

In this study, we characterized features of Tγδ LGLL, a rare and not yet well described T-LGL disorder. Through NGS analysis we found that Tγδ LGLL clonotypes are diversified between symptomatic and asymptomatic patients. We also demonstrated the importance of a combined evaluation of immunophenotype and *STAT* mutational profile for the correct management of patients presenting with Tγδ cell expansions.

CDR3 deep analysis, obtained both on gamma and delta TCR chains, provided evidence that the lymphoproliferation of clonal cells in Tγδ LGLL can include more than one unique clone, as already reported in Tαβ LGLL by several studies^22,36,37^, and is characterized mainly by public clonotypes. This observation supports and extends a previous study on the effector cells of elderly people showing that the dominant clonotypes exhibited by 2 cases of Tγδ LGLL were public, suggesting that leukemic Tγδ LGLs originate from a normal repertoire^38^. These features emphasize the difference with CD8+ Tαβ LGLL, in which Clemente et al.^22^ identified a decreased diversity of the TCR repertoire and showed that T-LGL clonotypes can be private within the disease, and CD4+ Tαβ LGLL, recently described as characterized predominantly by T-LGL clonotypes private to the patients^23^. In Tγδ LGLL, excluding that clonal specificity might be private to the disorder, we hypothesize that clonal LGLs do not arise under the pressure of neo-antigens or unique exogenous antigenic drivers, but rather serve to recognize auto or viral antigens. However, no relationships were found between the clonotypes reported in the literature as a consequence of activation by pathogens, i.e., CMV or TBC, and the clonotypes we detected in our patients. A possible explanation rests on the fact that only a very limited number of Tγδ ligands and structures have been defined so far.

Despite their public nature, our results demonstrated that most of the leukemic immunodominant gamma clonotypes resulted more recurrent among the TCR repertoire of patients (both Tγδ LGLL and HSTCL) than healthy controls. However, the comparison has the potential limit that T cells from controls presumably correspond to different maturation stages, whereas tumor cells from Tγδ LGLL are skewed toward cytotoxic terminal T effector cells. In fact, their Tγδ expansions almost lack CD28 and CD62L, and are frequently characterized by the peculiar co-expression of CD45RA and CD45RO. Moreover, since NGS specificity at low frequencies can be reduced, further studies are needed to confirm the findings about the public or private nature of Tγδ leukemic clones. To overcome NGS-based approaches’ limits, some strategies are currently arising, such as ultrasensitive immune repertoire sequencing using unique molecular identifiers able to increase the quantification accuracy for low-frequency clones. To screen the TCR repertoire of Tγδ cells, this method has been recently developed for the rearrangement of TCR delta locus^39^.

In line with the similar TCR repertoire among patients and once again in contrast with Tαβ LGLL, we found that some Tγδ LGLL patients shared the same productive immunodominant/codominant CDR3 clonotypes of the gamma chain. Interestingly, the common clonotypes were found within the group of asymptomatic patients who present similar features. In fact, the clonal Tγδ cells showed the preferential usage of Vγ9-JγP gene and Vδ2-Dδ3-Jδ1 recombination. Consistently, these Tγδ expansions exclusively expressed Vδ2. Since normal circulating Tγδ lymphocytes mostly express Vδ2/Vγ9, coupled to the evidence that the majority of these cells use the JγP joining segment^40^, our results indicate that leukemic cells in asymptomatic patients arise from a most common repertoire, where sharing of dominant Vγ chains occurs more often, in particular among the more “simple” or semi-invariant Vγ9-JγP chains. On the other side, patients with more severe diseases (either T-LGLL or HSTCL) exhibited clonal Tγδ cells characterized by the absence of the Vδ2 determinant as well as various and less common Vγ-Jγ and Vδ-Dδ-Jδ gene combinations. This phenotype suggests that in symptomatic cases the clonotypes, even if public, are mostly peculiar and arise from a subset of cells less represented in physiologic conditions. According to these data, we can hypothesize that a different pathogenetic mechanism or a different kind of antigen is involved in Tγδ LGLL patients with distinct clinical evolutions. This hypothesis is consistent with the evidence that human Vγ9+/Vδ2+ T cells mainly identify microbial infections, while Vδ2 negative T lymphocytes are usually recruited for defence against viruses or tumors^41^. Besides their specificity for relevant antigens, distinct kinds of Tγδ cell expansion might be also related to their development in distinct tissue compartments. In fact, discrete gamma chains or CDR3 parts are able to recognize specific BTN/BTNL proteins rather than a direct antigen, as recently demonstrated^1^. BTNL surface proteins are enriched in specific cells (epithelial or tumoral)^1–3^ where they behave as antigen-presenting molecules, supporting the notion that different TCR gamma chains can denote different microenvironmental tissue affinities.

Even if both NGS and flow data are consistent in distinguishing symptomatic and indolent diseases, our results highlight that immunophenotype evaluation is worthwhile to establish the patient’s clinical features, thus envisaging the Vδ2/Vγ9 expression, easily detected by flow analysis, as a relevant clinical marker in Tγδ lymphoproliferation. In fact, a Vδ2+/Vγ9+ LGL expansion most often indicates (19/21 patients in our series, Table 2) an asymptomatic clinical course, while Vδ2− clonotypes characterize symptomatic patients.

The crucial role of the phenotype to distinguish patients with different clinical behavior was also demonstrated by evaluating CD5 and CD56, more frequent in indolent Tγδ LGLL and uncommon in symptomatic patients. In addition, confirming our previous data collected on 10 Tγδ LGLL cases^42^, we observed that NKG2A expression was detected in 74% of asymptomatic patients and was absent in symptomatic ones. At variance, NKG2C activating haplotype was associated with the aggressive behavior of the HSTCL and is found only among symptomatic Tγδ LGLL.

Finally, we herein show a correlation among *STAT* genotype, immunophenotype, and clinical data. *STAT3* somatic mutations were consistently found in Tγδ patients with CD56− LGLs and symptomatic disease, whereas patients with CD56+ LGLs presented *STAT5B* mutations and indolent clinical course. Similarly, in Tαβ LGLL it has been previously shown that *STAT3* mutated patients belonged to the subgroup of CD8+ T-LGLL CD16+/CD56− presenting a symptomatic clinical course, while CD4+ T-LGLL CD56+ cases were asymptomatic with wild type *STAT3* but frequently mutated *STAT5B*^18,43,44^. The association between CD56 expression and *STAT5B* mutations has been confirmed also in the 3 HSTCL included in this paper, even if these two factors correlated with two different clinical courses, indolent in Tγδ LGLL and aggressive in HSTCL. Similarly to HSTCL, *STAT5B* mutation^19^ and CD56 expression^45^ can characterize the very rare aggressive form of CD8+ Tαβ LGLL. Hence, while clinical implications of the *STAT5B* mutations are still ambiguous and are likely to be not strong enough to define the disease course, we confirmed in Tγδ LGLL, as previously reported in Tαβ LGLL^18^, that *STAT3* mutations characterize neutropenic patients, supporting the hypothesis we recently proposed that the activation of *STAT3* accounts for the molecular mechanisms, related to the miR-146b/Fas ligand axis^46^, that ultimately lead to neutropenia.

In summary, our data highlight the importance of a combined evaluation of immunophenotype and *STAT* mutational profile for the correct management of patients with Tγδ cell expansions, as depicted in the Supplementary Fig. 4. Furthermore, our study emphasizes similarities and differences of Tγδ LGLL as compared with Tαβ LGLL and HSTCL and offers an explanation regarding the different pathways leading to a symptomatic or to an indolent disease.

## Methods

### Patients

The entire cohort of the study included a total of 39 patients (36 Tγδ LGLL and 3 HSTCL), comprehensive of a pilot group of 9 Tγδ LGLL and 2 HSTCL, analyzed by NGS for clonotype repertoire and *STAT* mutations, and a larger retrospective cohort including 28 patients (27 Tγδ LGLL and 1 HSTCL) assessed for their immunophenotype, genetic status of *STAT3/STAT5B* mutations by Sanger sequencing and clinical course. The patients affected by HSTCL expressing TCRγδ were included in the study for comparison.

All patients have been recruited by the Hematology Unit of Padova Hospital since 2001 and retrospectively revised to meet 2017 WHO criteria for T-LGLL or HSTCL diagnosis. In all 39 cases, clonality was evaluated by standard fragment length analysis of TCRγ gene rearrangement in PBMC. The identification of T-cell clonality was performed by IdentiClone TCR γ gene Rearrangement Assay (Invivoscribe, San Diego, CA) using the software provided by the manufacturer.

The evaluated clinical features included the co-presence of autoimmune diseases and clinically relevant cytopenias, classified as anemia (hemoglobin, Hb < 120 g/L), neutropenia (absolute neutrophil count, ANC < 1.5 × 10^9^/L) and thrombocytopenia (platelet count, PLT < 100 × 10^9^/L). Being the cytopenias and/or constitutional symptoms the variables considered to treat for LGLL, we distinguished the patients in symptomatic and asymptomatic for the presence and the absence of cytopenias and/or constitutional symptoms, respectively. All the samples evaluated in this study were obtained from patients out of or pre-therapy.

For clonotype repertoire evaluation by NGS performed in the pilot group, a control group of 23 samples represented by healthy blood donors was included. The control group was characterized by age (median: 64, IQR: 51–70) and gender (M/F: 12/11) matching the values of the patients’ group (median age: 65, IQR: 55–74; M/F: 5/6).

This study was performed according to the Helsinki Declaration and patients and controls gave written informed consensus before their inclusion in the study. The study and blood sample collection were approved by the Padova University Hospital Ethics Committee (approval number 4213/AO/17).

### TCR repertoire deep sequencing

CDR3 repertoire and frequency distribution of TCR γ and δ gene rearrangements were studied in the main cohort by NGS with the Illumina MiSeq on PBMC isolated from patients and controls. To prepare NGS libraries from at least 50 ng of genomic DNA, we used LymphoTrack Dx TCRG Assay (Invivoscribe, San Diego, USA) for γ chain, whereas for δ chain, not being available a corresponding Invivoscribe assay, we used the standard operating procedure of Euroclonality-NGS working group (www.euroclonality.org/protocols/). The two methods were based on a multiplexed PCR method amplifying the DNA between primers that target the conserved V and J regions of antigen receptor genes. The amplicons generated were then purified, quantified, pooled, and loaded onto a flow cell for sequencing with an Illumina MiSeq sequencing platform. The median of all the obtained sequence reads was 408942 (IQR: 251849–713940.5) for γ chain, and 429308.5 (IQR: 384705–472484.25) for δ chain.

### Bioinformatics and statistical analysis

The alignment of sequencing reads on V, D, and J segments of TCR, assembly of aligned sequences into clonotypes, conversion from nucleotides into amino acid sequences, and computation of the sequencing counts were performed by MiXCR^47^. For both tumor and control tissue samples, the identification of functionality of the rearranged sequences and the CDR3 repertoire analyses were achieved by VDJtools^48^.

The TCRγ and TCRδ CDR3 region (clonotype) was defined according to the International Immunogenetics Information System (IMGT). The CDR3 repertoire analyses were limited to productive sequences (i.e., in-frame sequences not containing stop codons and able to produce a functional protein) including only functional genes (no open reading frame and pseudogenes).

The clonotypes with frequency ≥5%, within symptomatic and indolent disease group, were aligned using ClustalW with default parameters (https://www.genome.jp/tools-bin/clustalw) and then represented as sequence logos using WebLogo (https://weblogo.berkeley.edu/logo.cgi).

Amino acid sequences of clonotypes observed with frequency ≥5% in at least one patient, were used to search, by a custom-made Python script, 100% identical sequences amongst all the clonotype sequences (CDR3) observed in the whole set of patients and healthy donors. The frequency of a clonotype was deemed significantly different in patients compared with control samples using the non-parametric Wilcoxon test (*P* value < 0.05), as the frequency distribution evaluated by the Shapiro–Wilk test was not normal.

Comparisons of proportions, ranks, or averages of variables between groups were performed by the *χ*^2^ test or Fisher’s test elaborated through the GraphPad Prism 6 software. The results were expressed as means with standard mean error (SEM). A value of *P* < 0.05 was accepted as significant.

### Flow cytometric analysis

The frequency of LGLs positive for the characteristic antigens was assessed by flow cytometry analysis using direct immunofluorescence assay combining up to six colors. Cells were evaluated using a FACSCanto analyzer (BD Biosciences) and data processed by FACSDiva software program (Becton Dickinson). The investigation for LGL surface markers was performed on whole peripheral blood anticoagulated with EDTA and on purified PBMC. The commercially available mouse monoclonal antibodies used included antibodies from Becton Dickinson: CD3 APC (SK7, 5 μl/test, cod. 345767), CD3 PE-CY7 (SK7, 5 μl/test, cod. 341111), CD3 APC-CY7 (SK7, 5 μl/test, cod. 341110), CD4 FITC (SK3, 5 μl/test, cod. 345768), CD5 PE-CY7 (L17F12, 5 μl/test, cod. 348810), CD8 PE (SK1, 5 μl/test, cod. 345773), CD16 FITC (NKP15, 20 μl/test, cod. 335035), CD16 PERCP-CY5.5 (3G8, 20 μl/test, cod. 338440), CD28 PE (L293, 20 μl/test, cod. 348047), CD45RA PE-CY7 (L48, 20 μl/test, cod. 337186), CD45RO APC (UCHL1, 5 μl/test, cod. 340438), CD56 PE-CY7 (NCAM16.2, 5 μl/test, cod. 335826), CD56 APC (NCAM16.2, 5 μl/test, cod. 341027), CD57 FITC (333169, 20 μl/test, cod. 333169), CD62L PERCP (DREG-56, 20 μl/test, cod. 555545), CD94 FITC (HP-3D9, 20 μl/test, cod. 555888), HLA-DR PERCP (L243, 20 μl/test, cod. 347402), TCRγδ FITC (11F2, 20 μl/test, cod. 347903), TCRγδ PE (11F2, 20 μl/test, cod. 333141), CD158a FITC (HP-3E4, 20 μl/test, cod. 340531), CD158b PE (CH-L, 20 μl/test, cod. 559785), CD158e PE (DX9, 20 μl/test, cod. 340484); antibodies from R&D Systems: NKG2A PE (131411, 10 μl/test, cod. FAB1059P) and NKG2C APC (134591, 10 μl/test, cod. FAB138A); antibodies from Thermo Fisher Scientific: Vγ9 FITC (7A5, 5 μl/test, cod. TCR2720), Vδ1 FITC (TS8.2, 5 μl/test, cod. TCR2730) and Vδ2 FITC (15D, 5 μl/test, cod. TCR2732). The immunophenotype analysis and the gate strategy is shown in a representative patient as Supplementary Fig. 5.

### *STAT3/STAT5B* mutations analysis

In the pilot cohort (*n* = 11 cases, 9 Tγδ LGLL and 2 HSTCL) targeted NGS for the SH2 domain of *STAT3* (exons 20-21) and for SH2 and transactivation domains of *STAT5B* genes (exons 15–19) was performed on an Illumina MiSeq instrument following the TruSeq Custom Amplicon Assay (Illumina) pipeline from 50 ng of starting genomic DNA, which was taken from the same sample used for TCR-NGS analysis. Median sequencing depth was 5617X (IQR: 1545–12507X) for *STAT3* and 6996X (IQR: 3816–12075X) for *STAT5B*. Somatic variants were identified using the Illumina Variant Studio software. Variant validation was obtained by Sanger sequencing.

In the expanded cohort of patients, including additional 28 cases, *STAT3* and *STAT5B* mutations were evaluated by Sanger sequencing using the set of primers reported in literature^17,19^ in order to amplify the hot spot regions for the most common mutations (exons 20–21 for *STAT3* and exons 16–18 for *STAT5B*). The analysis was performed on PBMC (*n* = 17) or on purified Tγδ LGLs (*n* = 11) obtained using magnetic separations over columns (MACS; Miltenyi Biotec, Auburn, CA) with magnetic Micro-Beads coated with monoclonal anti-human antibodies against Tγδ (Miltenyi Biotec). Sanger sequencing was performed on DNA extracted from 1−20 × 10^6^ cells using the Puregene Cell Kit Plus (Qiagen, Milan) and then sequenced using dye terminator technology and an ABI 3130 sequencer (Applied Biosystems). Sequences were analyzed using Chromas Pro and Blast programs.

### Reporting summary

Further information on research design is available in the Nature Research Reporting Summary linked to this article.

## Supplementary information

Supplementary Information

Reporting Summary

## Data Availability

TCR sequencing data and *STAT3* and *STAT5B* amplicon sequencing data included in this study are available in a BioProject (PRJNA715076) in NCBI SRA database. The remaining data generated in this study are provided in the Article, Supplementary Information and Source Data file.
