# Supplementary material for: Defining TCRγδ lymphoproliferative disorders by combined immunophenotypic and molecular evaluation
Source: Nat Commun. 2022 Jun 8;13:3298. doi: 10.1038/s41467-022-31015-x (PMC9177852; doi:10.1038/s41467-022-31015-x)
Supplement: Supplementary file 1 — Supplementary Information [file 41467_2022_31015_MOESM1_ESM.pdf]

Supplementary Figures and Tables for:

Defining TCRγδ lymphoproliferative disorders by combined immunophenotypic and molecular evaluation

Antonella Teramo[1,2], Andrea Binatti[3], Elena Ciabatti[4], Gianluca Schiavoni[5], Giulia Tarrini[4], Gregorio Barilà[1,2], Giulia Calabretto[1,2], Cristina Vicenzetto[1,2], Vanessa Rebecca Gasparini[1,2], Monica Facco[1,2], Iacopo Petrini[6], Roberto Grossi[7], Nadia Pisanti[7], Stefania Bortoluzzi[3,8], Brunangelo Falini[5], Enrico Tiacci[5], Sara Galimberti[4], Gianpietro Semenzato[1,2]*, and Renato Zambello[1,2]*

[1]Department of Medicine (DIMED), Hematology and Clinical Immunology Branch, Padova University School of Medicine, Italy;
[2]Veneto Institute of Molecular Medicine (VIMM), Padova, Italy;
[3]Department of Molecular Medicine, University of Padova, Padova, Italy;
[4]Department of Clinical and Experimental Medicine, Section of Hematology, University of Pisa, Italy;
[5]Institute of Hematology and Center for Hemato-Oncology Research, University and Hospital of Perugia, Italy;
[6]Department of Translational Research on New Technologies in Medicine and Surgery, University of Pisa, Italy;
[7]Department of Computer Science, University of Pisa, Italy;
[8]CRIBI Biotechnology Centre, University of Padova, Padova, Italy.

***** These authors jointly supervised this work

Co-Corresponding authors:
Renato Zambello, M.D.
Department of Medicine, University of Padova
Via Giustiniani, 2 - 35128, Padova, Italy.
Phone +39 049 821 8651
Fax +39 049 821 1970
Email: r.zambello@unipd.it

Gianpietro Semenzato, M.D.
Department of Medicine, University of Padova
Via Giustiniani, 2 - 35128, Padova, Italy.
Phone +39 049 821 2298
Fax +39 049 821 1970
Email: g.semenzato@unipd.it

Supplementary Figure 1

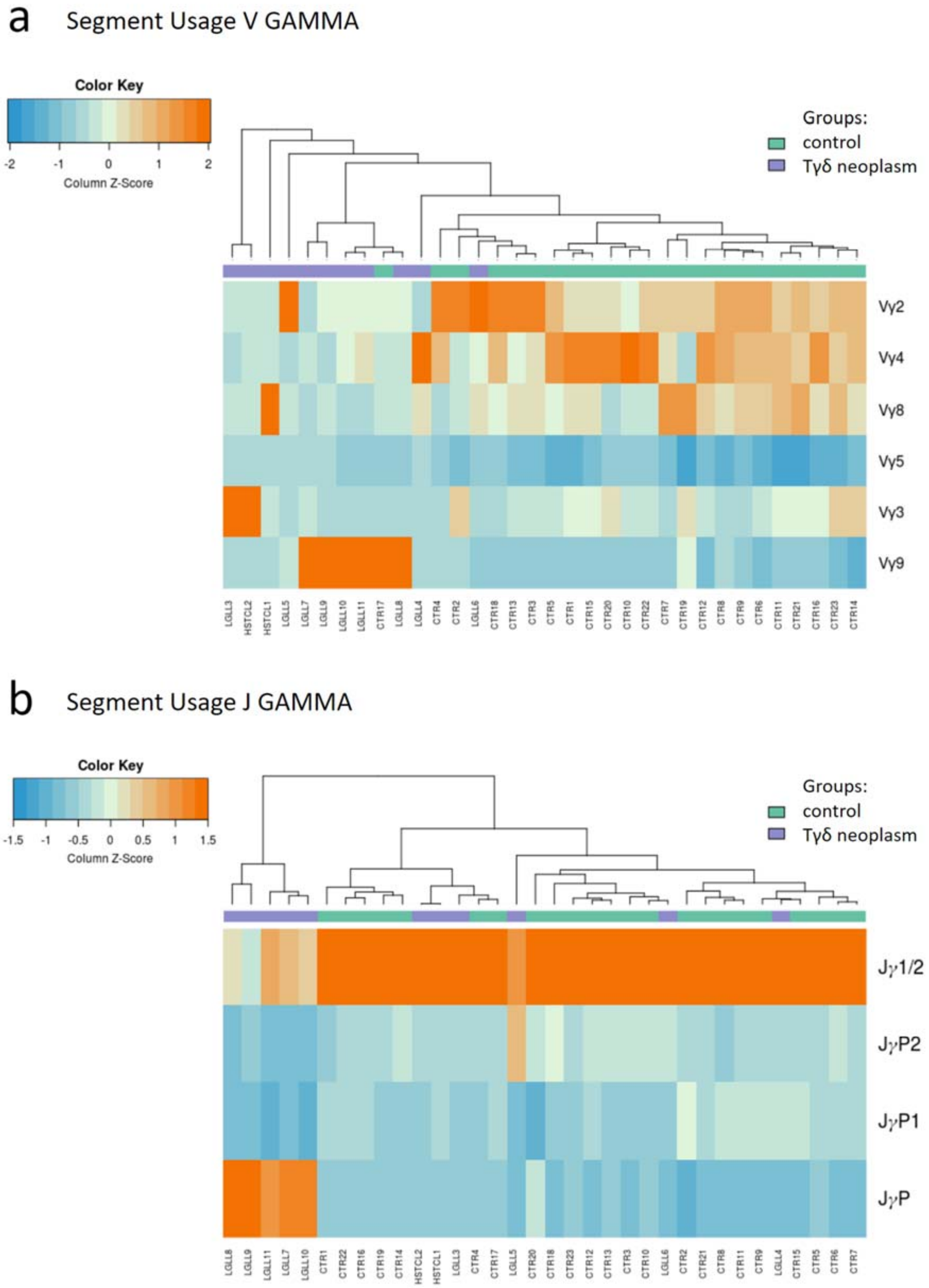

Supplementary Figure 1. Preferential V-J segment usage of the gamma chain in the entire TCR repertoire in patients (n=11, violet) and healthy controls (n=23, green). Sample clustering based on Variable segment usage of V (**a**) and J (**b**) genes. Weighted Variable usage profiles are used, hierarchical clustering is performed using Euclidean distance. Z-scores indicate the relative frequency of each segment. Dendrograms show clustering of the samples.

Supplementary Figure 2

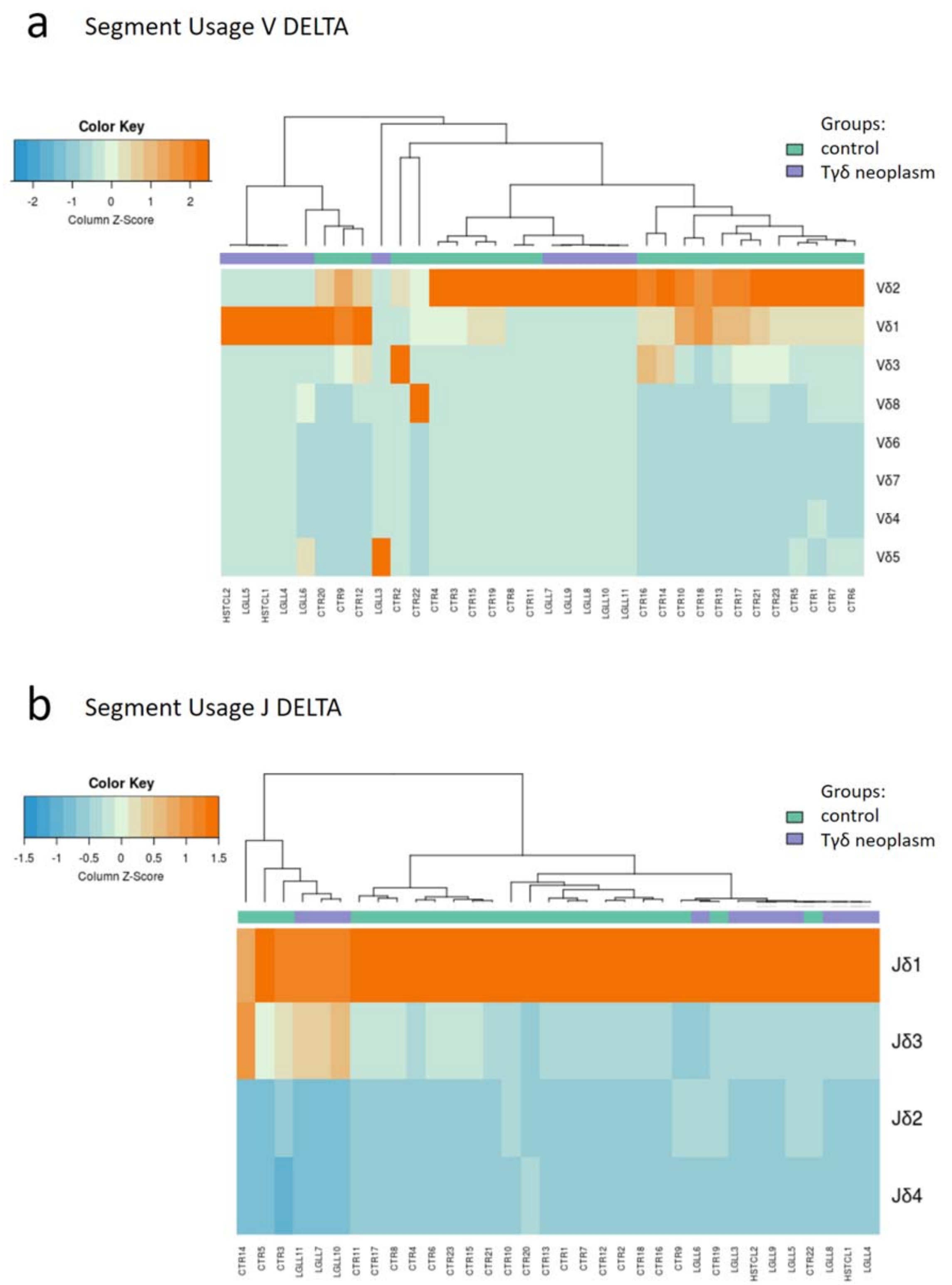

Supplementary Figure 2. Preferential V-J segment usage of the delta chain in the entire TCR repertoire in patients (n=11, violet) and healthy controls (n=23, green). Sample clustering based on Variable segment usage of V (**a**) and J (**b**) genes. Weighted Variable usage profiles are used, hierarchical clustering is performed using Euclidean distance. Z-scores indicate the relative frequency of each segment. Dendrograms show clustering of the samples.

Supplementary Figure 3

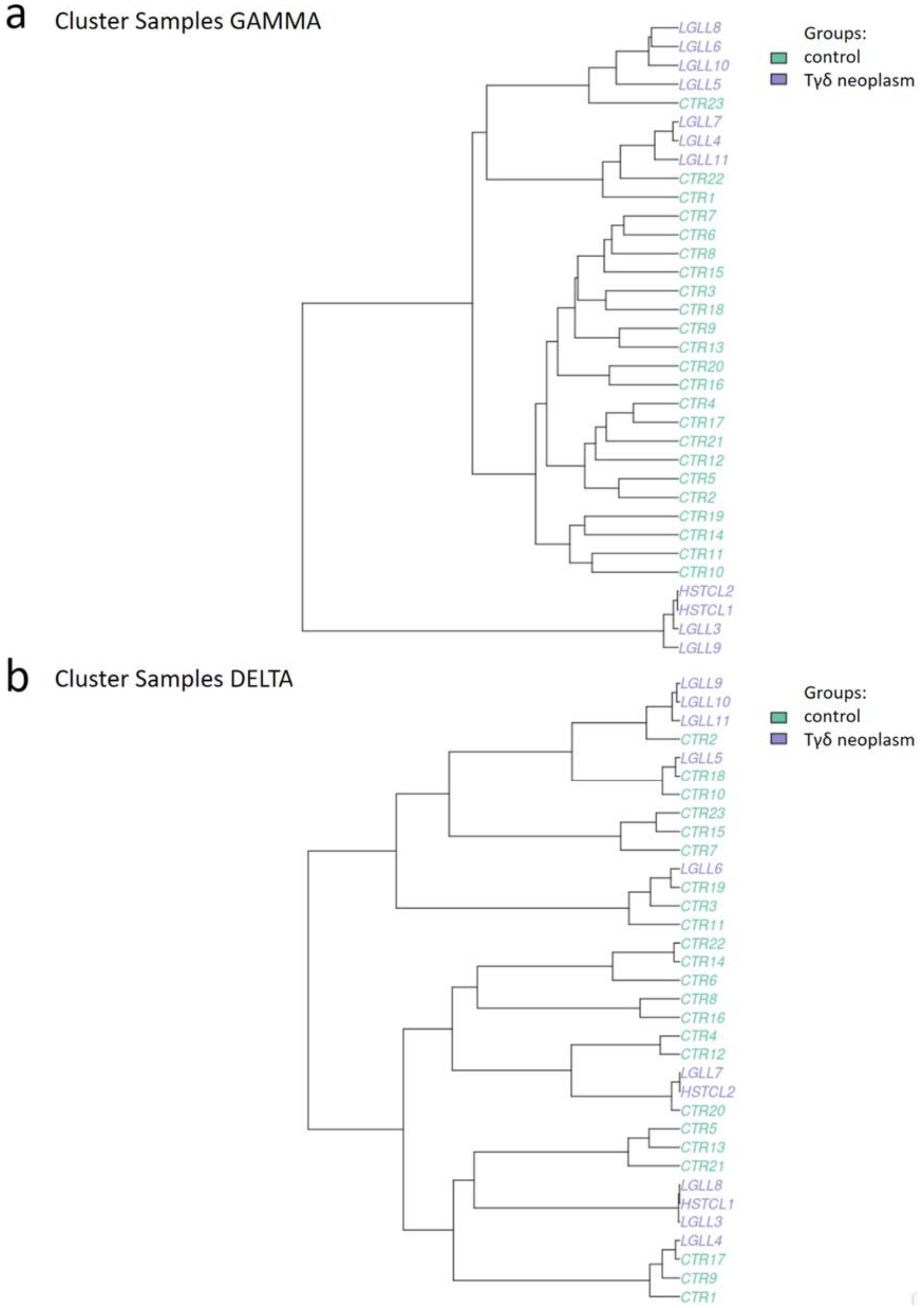

Supplementary Figure 3. Overlap and clustering of TCR repertoires. Hierarchical clustering of TCR repertoire of gamma chain (**a**) and delta chain (**b**) of patients (n=11, violet) and healthy controls (n=23, green). Branch length shows the distance between repertoires using the F pairwise similarity metric (the geometric mean of inter-sample overlap).

Supplementary Figure 4

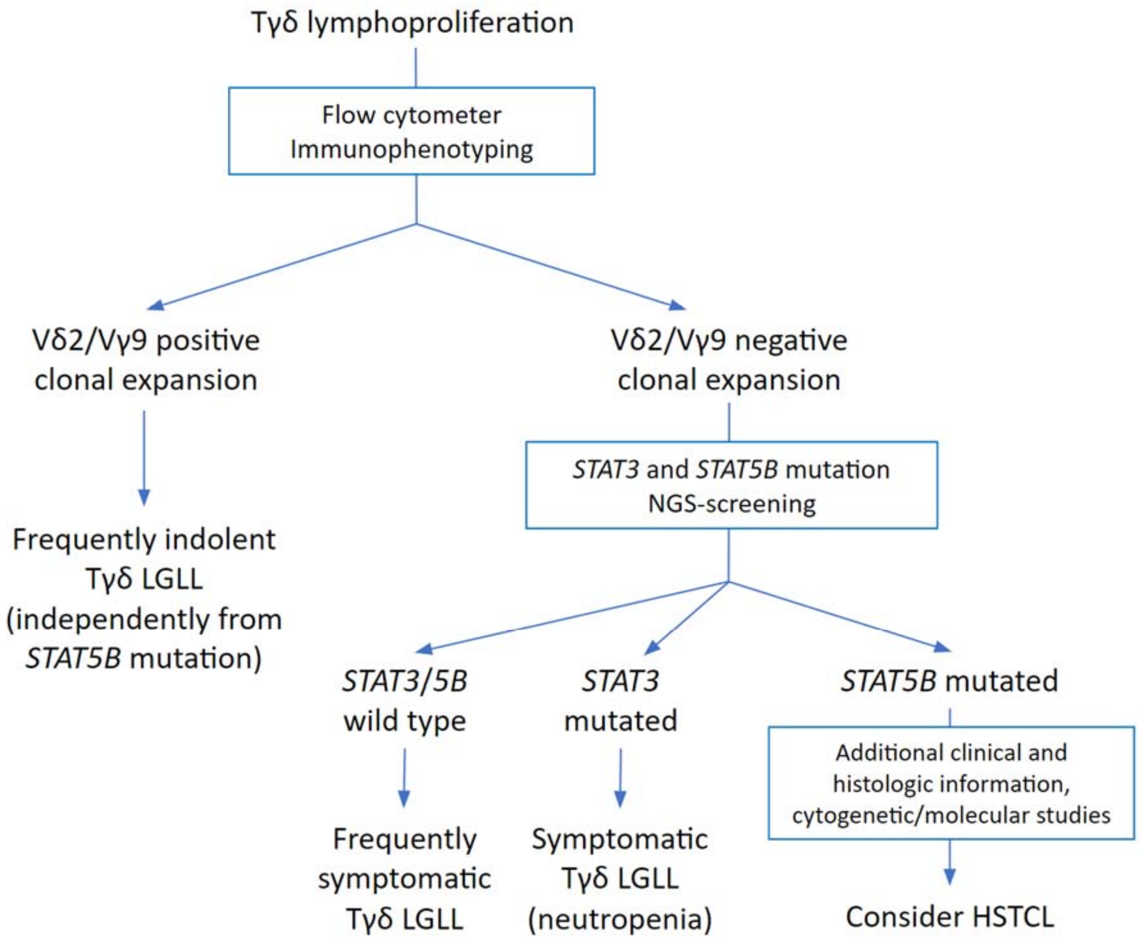

Supplementary Figure 4. Flow-chart of the immunophenotyping and mutational assessments we suggest to characterize patients affected by TCRγδ neoplasms. In the majority of cases, a Vδ2+/Vγ9+ LGL expansion is suggestive of an asymptomatic clinical course (independently from *STAT5B* mutations), while Vδ2-/Vγ9- clonotypes characterize symptomatic patients, neutropenic if carrying *STAT3* mutations. Conversely, the presence of *STAT5B* mutations in Tγδ clone not expressing Vδ2/Vγ9, particularly when CD57 expression is lacking, suggests considering HSTCL for the differential diagnosis and requests additional clinical and histologic findings, cytogenetic tests (detecting isochromosome arm 7q), and a more detailed immunophenotype analysis searching for indicators of immature cytotoxic T-cells (TIA1+ granzyme B- perforin-). *STAT3* and *STAT5B* gene analysis by NGS allow to also detect low burden mutations, within and outside the SH2 domain.

Supplementary Figure 5

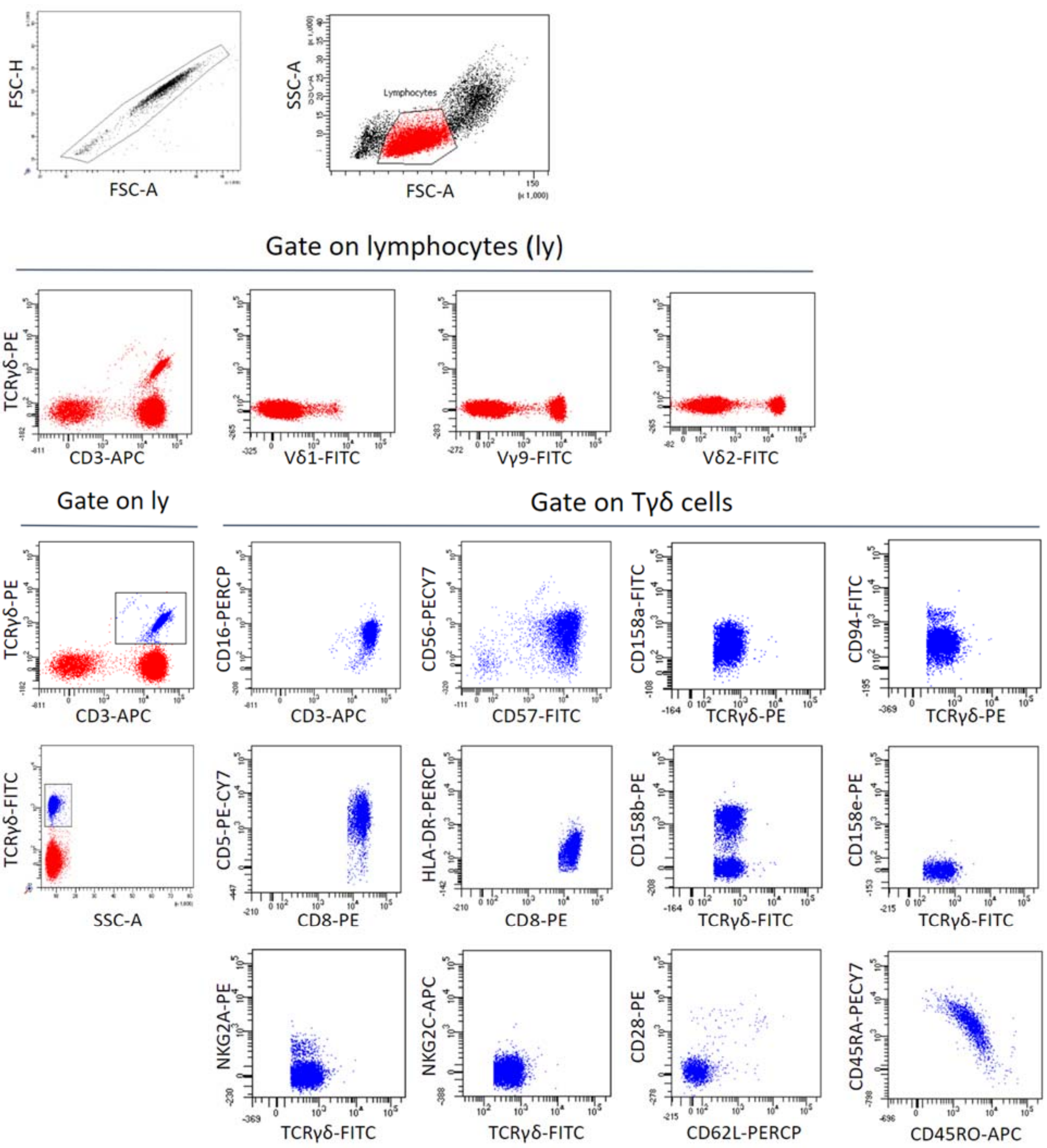

Supplementary Figure 5. Gating strategy and immunophenotypic characterization of Tγδ cell population in a representative patient affected by TCRγδ neoplasm. FSC-H vs FSC-A allowed to exclude doublets. Then, in the SSC-A vs FSC-A plot a morphologic gate was designed to identify lymphocytes within peripheral blood mononuclear cells (PBMC). PBMC were stained with CD3-APC, TCRγδ-PE, Vδ1-FITC, Vδ2-FITC and Vγ9-FITC to identify and characterize the percentage of Tγδ cells on lymphocytes. CD3+/Tγδ cell markers (CD5, CD8, HLA-DR, CD16, CD28, CD45RA, CD45RO, CD56, CD57, CD62L, CD94, CD158a, CD158b, CD158e, NKG2A, NKG2C) were determined by staining cells using two different anti-TCRγδ antibodies, i.e. TCRγδ FITC and TCRγδ PE, according to the multicolor flow cytometry panel design.

Supplementary Table 1. CDR3γ sequences > 5%. All the TCRγ clonotypes >5% from each patient with Tγδ disorders are shown. Nucleotides of each region identified in a junction are displayed: Vγ and Jγ gene segments, palindromic (P, blue) and non-templated (N, red) nucleotides added during recombination. Dots represent trimmed nucleotides. CDR3 amino acid sequences with the corresponding % of reads are reported.

Patients	V-gene	J-gene	3'V-REGION	N/P	5'J-REGION	Amino acid sequence	% total reads
**pt1**	Vγ8	Jγ1/2	tgtgccacctgggatag.	ttcg	....tattataagaaactcttt	CATWDSSYYKKLF	97.38%
**pt2**	Vγ3	Jγ1/2	tgtgccacctgggacagg	ttgc	..attattataagaaactcttt	CATWDRLHYYKKLF	98.20%
**pt3**	Vγ3	Jγ1/2	tgtgccacctgggacag.	a	.......tataagaaactcttt	CATWDRYKKLF	83.82%
**pt4**	Vγ4	Jγ1/2	tgtgccacctggga....	cgggcctagcatgg	..attattataagaaactcttt	CATWDGPSMDYYKKLF	61.61%
	Vγ8	JγP1	tgtgccacctgggatag.	aggggg	.taccactggttggttcaagatattt	CATWDRGGTTGWFKIF	18.89%
	Vγ3	JγP2	tgtgccacctgggacagg	ccc	........gattggatcaagacgttt	CATWDRPDWIKTF	5.63%
**pt5**	Vγ2	JγP2	tgtgccacctgggacggg	ccgggg	..agtagtgattggatcaagacgttt	CATWDGPGSSDWIKTF	40.99%
	Vγ2	Jγ1/2	tgtgccacctgggacggg	ccgtcg	....tattataagaaactcttt	CATWDGPSYYKKLF	35.66%
	Vγ8	Jγ1/2	tgtgccacctgggatagg	tgg	....tattataagaaactcttt	CATWDRWYYKKLF	8.85%
**pt6**	Vγ2	Jγ1/2	tgtgccacctgggacgg.	ccgagtt	.aattattataagaaactcttt	CATWDGRVNYYKKLF	25.11%
	Vγ2	JγP2	tgtgccacctgggac...	tatt	...gtagtgattggatcaagacgttt	CATWDYCSDWIKTF	9.18%
	Vγ4	Jγ1/2	tgtgccacctggga....	gaaggggaaattact	......ttataagaaactcttt	CATWEKGKLLYKKLF	7.12%
	Vγ8	JγP2	tgtgccacctgggatag.		....tagtgattggatcaagacgttt	CATWDSSDWIKTF	6.40%
**pt7**	Vγ9	JγP	tgtgccttgtgggaggtg	g	.....aagagttgggcaaaaaaatcaaggtattt	CALWEVEELGKKIKVF	32.04%
	Vγ9	Jγ1/2	tgtgccttg.........	gagagggg	............gaaactcttt	CALERGKLF	26.05%
	Vγ9	JγP	tgtgccttgtgggaggtg	cg	......agagttgggcaaaaaaatcaaggtattt	CALWEVRELGKKIKVF	22.93%
**pt8**	Vγ9	JγP	tgtgccttgtgggaggtg	cg	......agagttgggcaaaaaaatcaaggtattt	CALWEVRELGKKIKVF	55.65%
**pt9**	Vγ9	JγP	tgtgccttgtgggagg..	atcg	......agagttgggcaaaaaaatcaaggtattt	CALWEDRELGKKIKVF	61.34%
**pt10**	Vγ9	JγP	tgtgccttgtgggaggtg	cgt	.......gagttgggcaaaaaaatcaaggtattt	CALWEVRELGKKIKVF	20.75%
	Vγ9	JγP	tgtgccttgtgggagg..		.....aagagttgggcaaaaaaatcaaggtattt	CALWEEELGKKIKVF	19.02%
	Vγ9	JγP	tgtgccttgtggga....	tac	......agagttgggcaaaaaaatcaaggtattt	CALWDTELGKKIKVF	12.16%
**pt11**	Vγ9	JγP	tgtgccttgtgggaggtg	g	.....aagagttgggcaaaaaaatcaaggtattt	CALWEVEELGKKIKVF	36.53%

Supplementary Table 2. CDR3δ sequences > 5%. All the TCRδ clonotypes >5% from each patient with Tγδ disorders are shown. Nucleotides of each region identified in a junction are displayed: Vδ, Dδ and Jδ segments, palindromic (P, blue) and non-templated (N, red) nucleotides added during recombination. Dots represent trimmed nucleotides. CDR3 amino acid sequences with the corresponding % of reads are reported.

Patients	V-gene	D-gene	J-gene	3'V-REGION	N/P	D1-REGION	N/P	D2-REGION	N/P	5'J-REGION	Amino acid sequence	% total reads
**pt1**	Vδ1	Dδ3	Jδ1	tgtgctcttg.......	ccctccc	....ggggatacg	ggggt			acaccgataaactcatcttt	CALALPGIRGYTDKLIF	99.52%
**pt2**	Vδ1	Dδ2	Jδ1	tgtgctcttgggga...	tgatatccaccct	.cttcc...	aa			acaccgataaactcatcttt	CALGDDIHPLPNTDKLIF	95.05%
**pt3**	Vδ5	Dδ3	Jδ1	tgtgcagcaagcg	cg	...atag.	ggtccc	....gggga....		..accgataaactcatcttt	CAASAIGSRGTDKLIF	93.23%
**pt4**	Vδ1	Dδ3	Jδ1	tgtgctcttggggaa..	gcccc	actgggggatacg	catt			...ccgataaactcatcttt	CALGEAPLGDTHSDKLIF	92.98%
**pt5**	Vδ1	Dδ2	Jδ1	tgtgctcttggggaact	ggtagggggc	ccttc....	a			acaccgataaactcatcttt	CALGELVGGPFNTDKLIF	51.01%
	Vδ1	Dδ3	Jδ1	tgtgctcttggggaac.	ggag	...gggggatacg	tttggtg			...ccgataaactcatcttt	CALGERRGDTFGADKLIF	32.43%
	Vδ1	Dδ3	Jδ1	tgtgctcttggggaac.	cccc	...tccta.	gt	.ctggggga....	gtcg	........aaactcatcttt	CALGEPPPSLGESKLIF	14.97%
**pt6**	Vδ5	Dδ3	Jδ1	tgtgcagcaa...		.cttcct..	cgt	actgggggat...	ct	acaccgataaactcatcttt	CAATSSYWGIYTDKLIF	17.89%
	Vδ1	Dδ2	Jδ1	tgtgctcttgggg....	t	ccttcct..	ccgggcgtac			......ataaactcatcttt	CALGVLPPGVHKLIF	9.72%
	Vδ1	Dδ3	Jδ1	tgtgctcttggg.....	cccttccttcct	actgggggat...				acaccgataaactcatcttt	CALGPFLPTGGYTDKLIF	9.01%
	Vδ1	Dδ2	Jδ1	tgtgctcttggggaa..	gccgctccctaccag	ccttc....	gt			acaccgataaactcatcttt	CALGEAAPYQPSYTDKLIF	7.21%
	Vδ8	Dδ3	Jδ1	tgtgcttataggagc.	tcgacacta	..ttcc...	cgt	actgggggat...	tcggc	...ccgataaactcatcttt	CAYRSSTLFPYWGIRPDKLIF	6.68%
**pt7**	Vδ2	Dδ3	Jδ1	tgtgcctgtgacacc	tt	actgggggatacg	cgtagta			acaccgataaactcatcttt	CACDTLLGDTRSNTDKLIF	60.70%
	Vδ2	Dδ3	Jδ3	tgtgcctgtgacacc	ct	actgggggatacg	gagga			ctcctgggacacccgacagatgtttttc	CACDTLLGDTEDSWDTRQMF	38.88%
**pt8**	Vδ2	Dδ3	Jδ1	tgtgcctgtgacac.	gg	....tagt	tag	...gggggat...	ctca	acaccgataaactcatcttt	CACDTVVRGDLNTDKLIF	98.18%
**pt9**	Vδ2	Dδ3	Jδ1	tgtgcctgtgacacc	gtcggt	.ctgggggatacg	ccgtca			..accgataaactcatcttt	CACDTVGLGDTPSTDKLIF	82.50%
	Vδ2	Dδ3	Jδ1	tgtgcctgtgac...	gt	actgggggatacg				..accgataaactcatcttt	CACDVLGDTTDKLIF	5.5%
**pt10**	Vδ2	Dδ3	Jδ3	tgtgcctgtgacacc	tcc	...gggga....	caccctct			ctcctgggacacccgacagatgtttttc	CACDTSGGHPLSWDTRQMFF	39.73%
	Vδ2	Dδ3	Jδ1	tgtgcctgtgacacc	gtaggg	.ctggggga....	aaacgggg			...ccgataaactcatcttt	CACDTVGLGENGADKLIF	37.66%
	Vδ2	Dδ3	Jδ1	tgtgcctgtgac...	tcaat	actgggg.....	ccctccggcgatccccga			acaccgataaactcatcttt	CACDSILGALRRSPNTDKLIF	16.96%
**pt11**	Vδ2	Dδ3	Jδ1	tgtgcctgtgacacc	gtagaggga	..tgggggatac.	aggcggg			....cgataaactcatcttt	CACDTVEGWGIQAGDKLIF	39.19%
	Vδ2	Dδ3	Jδ3	tgtgcctgtgac...	tct	actgggg......	agat			ctcctgggacacccgacagatgtttttc	CACDSTGEISWDTRQMFF	31.07%
	Vδ2	Dδ3	Jδ1	tgtgcctgtgacac.		actgggggat...				..accgataaactcatcttt	CACDTLGDTDKLIF	7.25%
	Vδ2	Dδ3	Jδ3	tgtgcctgtgacacc	gttcgg	actgggggatacg				..cctgggacacccgacagatgtttttc	CACDTVRTGGYAWDTRQMFF	5.40%

Supplementary Table 3. Source Data of Figure 2a (Recurrence of gamma and delta chain immunodominant CDR3 sequences of γδ disease patients) of the Main Manuscript. The values in the matrix indicate the frequency percentage of the clonotype sequences (first column) in sample TCRγ repertoires of the 11 Tγδ neoplasia patients and 23 healthy controls (listed in the first row). The first 12 gamma clonotypes listed are significantly more frequent in patients than in controls. The P-value is reported in the last column (non-parametric Wilcoxon test, one-sided, * P<0.05).

	CTR1	CTR2	CTR3	CTR4	CTR5	CTR6	CTR7	CTR8	CTR9	CTR10	CTR11	CTR12	CTR13	CTR14	CTR15	CTR16	CTR17	CTR18	CTR19	CTR20	CTR21	CTR22	CTR23	HSTCL1	HSTCL2	LGLL3	LGLL4	LGLL5	LGLL6	LGLL7	LGLL8	LGLL9	LGLL10	LGLL11	P Value
CATWDGPSMDYYKKLF	0.001186	0.020004	0	0	0	0	0	0	0	0	0	0	0	0	0	0	0.003144	0	0	0	0	0.009466	0	0.002012	0.011422	0.005003	61.61402	0.046579	0.003897	0.026039	0.144503	0.001532	0.019552	0.026117	1.94e-06
CALERGKLF	0.005934	0	0	0	0.016130	0	0	0	0	0	0	0	0	0	0	0	0	0	0	0	0	0	0	0.001078	0.003263	0.000834	0.004552	0	0.001558	26.05284	0.024083	0.004597	0.009776	0.007462	8.98e-06
CATWDSSYYKKLF	0	0	0	0	0	0.023739	0	0.067702	0	0	0	0	0.244099	0	0	0	0	0	0.884725	0	0	0	0	97.38904	0.119115	0.092571	0.074656	0.155263	0.067422	0.031826	0.202993	0.058240	0.109166	0.134318	1.51e-05
CATWEKGKLLYKKLF	0	0	0	0	0	0	0	0	0	0	0	0	0	0	0	0	0	0	0	0	0	0	0	0.000107	0	0	0.000910	0	7.121867	0.000180	0.003440	0.001532	0.001629	0	1.54e-05
CATWDYCSDWIKTF	0	0	0	0	0	0	0	0	0	0	0	0	0	0	0	0	0	0	0	0	0	0	0	0.000395	0	0	0.001820	0.010350	9.189758	0	0	0.006130	0.001629	0	7.41e-05
CATWDRGGTTGWFKIF	0	0.001538	0	0	0	0	0	0	0	0	0	0	0	0	0	0	0	0	0	0	0	0	0	0.000215	0	0.001668	19.89038	0	0.000389	0.002531	0.010321	0	0	0.003731	8.43e-05
CALWEDRELGKKIKVF	0	0	0	0	0	0	0	0.003563	0	0	0	0.001550	0	0	0	0	0	0	0	0	0.005149	0	0	0.000898	0.003263	0.001668	0.002731	0	0.001169	0	0.233958	61.34045	0.006517	0	4.47e-04
CATWDRLHYYKKLF	0	0	0	0	0	0	0	0.022567	0	0	0	0	0	0	0	0	0	0	0	0	0.200848	0	0.163624	0.000682	98.20347	0.003335	0.005462	0	0.005845	0.000542	0.061930	0.001532	0	0.003731	4.52e-04
CALWDTELGKKIKVF	0	0	0	0	0	0	0	0	0	0	0	0	0	0	0	0	0	0	0	0	0	0	0	0	0	0	0.001820	0	0.000389	0	0	0	12.16150	0	5.49e-03
CATWDGRVNYYKKLF	0	0	0	0	0.025348	0	0	0	0	0	0	0	0	0	0.005084	0	0	0	0	0	0.032616	0	0.001022	0.000575	0	0	0	0.005175	25.11087	0.000180	0.003440	0.039848	0.016293	0	5.79e-03
CALWEVEELGKKIKVF	0.010681	0	0	0	0.041479	0.012293	0	0.030881	0	0	0	0	0.073901	0.001061	0.142994	0	0	0	0	0	0.046349	0.022088	0	0.001976	0.011422	0.021683	0.014567	0.015526	0.004287	32.03960	0	0.017625	0.013034	36.52712	0.01
CATWDRPDWIKTF	0	0	0	0	0	0	0	0	0	0	0	0	0	0	0	0	0.001572	0	0	0	0	0	0	0	0	0.002501	5.634713	0	0	0.000723	0	0	0	0	0.03
CATWDGPGSSDWIKTF	0	0	0	0.835807	0	0.069521	0.053117	0	0	0	0	0	0	0	0	0	0	0	0	0	0	0	0	0	0	0	0	40.99472	0.000779	0	0.051608	0	0	0.003731	not significant
CALWEEELGKKIKVF	0.001186	0	0	0	0	0.013989	0.004994	0.030287	0	0	0.030998	0	0	0	0.002542	0	0	0.020463	0	0.000651	0.050641	0.015777	0.422355	0.000107	0	0	0.000910	0	0.001169	0	0.010321	0.023756	19.02108	1.074546	not significant
CATWDRWYYKKLF	0	0	0	0	0	0	0	0	0	0	0	0	0	0	0	0	0	0	0	0	0.028324	0	0	0	0	0	0	8.855191	0	0	0	0	0	0	not significant
CALWEVRELGKKIKVF	0.005934	0	0	0.569556	0.109458	0.022043	0	0.105116	0.279495	0	0.152608	0	0.120929	0.115727	0.013981	0.206209	0	0.100270	0	0.379365	0.448045	0.011570	0.203507	0.017933	0	0.019181	0.032775	0.020701	0.005066	22.92718	55.64768	0	20.75145	0.007462	not significant
CATWDRYKKLF	0	0	0.336169	0.000529	0.033413	0.132685	0.086259	0.099178	0	0.232805	0.098956	0	0.017915	0	0	0.409473	0.149352	0.110501	0	0.105596	0.216297	0.123063	0	0.000970	0.006526	83.81912	0	0.098333	0.028060	0.014285	0.079133	0.004597	0.008146	0.052234	not significant
CATWDSSDWIKTF	0.002373	0	0.673642	0	0.504660	0.117848	0.087621	0.212015	0	0	0	0.000258	0.470282	0	0.084525	0	0.328575	0	0.327125	0	3.972327	0.141996	0	0	0.001631	0	0.011835	0	6.396196	0.014466	0.113538	0.045213	0	0	not significant
CATWDGPSYYKKLF	0.352488	0.081554	0	0.410228	0.076044	0.111489	0.095793	0.036226	0	0	0.048882	0	0	0	0	0.229776	0.091183	0	0	0	0	0.783608	0.314976	0.000215	0	0	0.006373	35.66400	0.016368	0	0	0	0.039104	0.003731	not significant

Supplementary Table 4. Source Data of Figure 2b (Recurrence of gamma and delta chain immunodominant CDR3 sequences of γδ disease patients) of the Main Manuscript. The values in the matrix indicate the frequency percentage of the clonotype sequences (first column) in sample TCRγ repertoires of the 11 Tγδ neoplasia patients and 23 healthy controls (listed in the first row). The first 2 delta clonotypes listed are significantly more frequent in patients than in controls. The P-value is reported in the last column (non-parametric Wilcoxon test, one-sided, * P<0.05).

	CTR1	CTR2	CTR3	CTR4	CTR5	CTR6	CTR7	CTR8	CTR9	CTR10	CTR11	CTR12	CTR13	CTR14	CTR15	CTR16	CTR17	CTR18	CTR19	CTR20	CTR21	CTR22	CTR23	HSTCL1	HSTCL2	LGLL3	LGLL4	LGLL5	LGLL6	LGLL7	LGLL8	LGLL9	LGLL10	LGLL11	P Value
CALGELVGGPFNTDKLIF	0	0.042537	0	0	0	0	0	0	0	0.017500	0	0	0	0	0	0	0	0.009626	0	0	0	0	0	0	0.000711	0.004083	0.002301	51.01496	0	0.001036	0	0	0	0	0.04
CALGDDIHPLPNTDKLIF	0	0	0	0.019372	0	0	0.048847	0.020910	0	0	0.001069	0.049012	0.001086	0	0.020225	0.019286	0	0.001132	0	0.025440	0	0.014229	0.054658	0.006444	99.75950	0.054713	0	0.006791	0.002523	0.038854	0.008486	0.000852	0.009026	0.020769	0.05
CALALPGIRGYTDKLIF	0	0	0	0.011531	0	0.034301	0	0.027706	0	0	0.002138	0.032675	0	0.015923	0	0.024546	0	0.001698	0.000276	0.023883	0	0.017193	0	99.51850	0	0.097994	0	0.003395	0.001261	0.019686	0.003916	0.011078	0.000668	0.020769	not significant
CALGEAPLGDTHSDKLIF	0.013617	0	0	0	0	0	0	0.017250	0.033975	0	0	0	0	0	0	0.007451	0.025512	0	0.000276	0	0	0	0	0.001841	0	0.023682	92.97543	0.001358	0	0.002072	0	0	0	0.012000	not significant
CAATSSYWGIYTDKLIF	0	0	0	0	0	0	0	0	0	0	0.005881	0	0	0	0	0	0	0	0.001656	0	0	0	0	0	0	0	0	0	17.89354	0	0.000652	0	0.000668	0	not significant
CALGERRGDTFGADKLIF	0	0.029449	0	0	0	0	0	0	0	0.009722	0	0	0	0	0.000577	0	0	0.003964	0	0	0	0	0	0	0	0	0	32.43054	0.001261	0.001554	0.001305	0	0	0	not significant
CACDTVVRGDLNTDKLIF	0.023830	0	0	0	0.037035	0	0	0	0.028192	0	0	0	0.022821	0	0	0	0.021685	0	0.000828	0	0.017564	0	0	0.007365	0	0.004083	0.023778	0	0.001892	0.002590	98.18066	0	0	0	not significant
CALGEAAPYQPSYTDKLIF	0	0	0.002324	0	0	0	0	0	0	0	0.002138	0	0	0	0	0	0	0	0.001380	0	0	0	0	0	0.000711	0	0	0.001358	7.211793	0	0	0	0	0	not significant
CAYRSSTLFPYWGIRPDKL	0	0	0	0	0	0	0	0	0	0	0.001069	0	0	0	0	0	0	0	0.001104	0	0	0	0	0.000920	0	0	0	0	6.681300	0	0	0	0	0	not significant
CACDTLLGDTRSNTDKLIF	0.030639	0.006544	0	0.007841	0.038094	0	0	0	0.030360	0	0	0.037834	0.028798	1.053689	0	0.001315	0.017858	0	0	0.020248	0.016639	0.004743	0.009331	0.011047	0.010673	0.011432	0.036818	0.002037	0.001892	60.69959	0.037209	0	0.001002	0	not significant
CAASAIGSRGTDKLIF	0.029277	0	0	0.025369	0.044443	0.005717	0.003117	0.016728	0.026023	0	0	0.059330	0.033145	0	0	0.018847	0.044009	0	0	0.029074	0.018951	0	0	0.013809	0.003557	93.22777	0.036818	0	0.001261	0.032638	0.030028	0	0	0.012461	not significant
CACDSTGEISWDTRQMFF	0	0.081803	0	0	0.004232	0.020581	0	0.011500	0	0.019444	0	0.001719	0	0.008785	0	0.007451	0	0.014723	0	0.000519	0	0.011857	0	0.022095	0	0.009799	0	0.008828	0	0	0	0.010225	0.001337	31.07406	not significant
CALGVLPPGVHKLIF	0	0	0.003098	0	0	0	0	0	0	0	0.003742	0	0	0	0	0	0	0	0.000828	0	0	0	0	0.001841	0	0	0	0	9.724850	0	0	0	0	0	not significant
CACDTVGLGDTPSTDKLIF	0.002723	0.170151	0.003873	0.003690	0.006349	0.066316	0	0	0	0.036296	0	0.000859	0	0.019767	0	0	0	0.024350	0	0	0	0.032015	0	0.044191	0	0	0	0.023090	0	0.001036	0	82.49545	0.000668	0.032769	not significant
CACDTLLGDTEDSWDTRQ	0.011574	0	0	0.009686	0.020105	0	0	0	0.023855	0	0	0.030095	0.013584	0	0	0	0.012118	0	0	0.015056	0.011555	0	0.005332	0	0.003557	0	0.019176	0.000679	0.002523	38.87662	0.028722	0	0	0	not significant
CACDTVEGWGIQAGDKLI	0.004085	0.085075	0.003873	0.000922	0.004232	0.024011	0	0.010455	0	0.023333	0	0	0	0.013727	0	0.005698	0	0.023784	0	0	0	0.013043	0	0.018413	0	0.013882	0	0.01562	0	0	0	0.012356	0.001002	39.19358	not significant
CALGEPPPSLGESKLIF	0	0.022905	0	0	0	0	0	0	0	0.004537	0	0	0	0	0	0	0	0.002265	0	0	0	0	0	0	0	0.000816	0	14.96533	0	0	0	0	0	0	not significant
CALGPFLPTGGYTDKLIF	0	0	0.001549	0	0	0	0	0	0	0	0.002673	0	0	0	0	0	0	0	0.001656	0	0	0	0	0	0	0	0	0	9.007014	0.000518	0	0	0	0	not significant
CACDTLGDTDKLIF	0	0	0	0	0	0.016007	0	1.321526	0	0.003888	0.140082	0	1.608915	0.004392	0	0.003506	0	0.002831	0	0	0.172408	0.004743	0	0.002761	0	0.024498	0	0.003395	0	0	0.001305	0.002556	0.000334	7.254584	not significant
CACDVLGDTTDKLIF	0	0.013088	0	0.001383	0	0.005717	0	0	0	0	0.000534	0	0	0.001647	0	0	0	0	0	0	0	0.002964	0	0.002761	0	0	0	0	0	0	0	5.498532	0	0.004153	not significant
CACDSILGALRRSPNTDKLI	0	0.035993	0.002324	0.000922	0.004232	0.021724	0.010393	0	0	0.009074	0	0	0	0.007687	0.008668	0	0	0.007361	0	0	0	0.011264	0.007332	0.015651	0.00925	0	0	0.004753	0	0	0	0.009373	16.96141	0	not significant
CACDTVGLGENGADKLIF	0.003404	0.111252	0.003098	0.000922	0.009523	0.048022	0.028061	0	0	0.019444	0	0	0	0.020316	0.015602	0	0	0.010193	0	0	0	0.019565	0.019330	0.020254	0.011384	0	0	0.017657	0	0	0	0.017043	37.65536	0.000923	not significant
CACDTSGGHPLSWDTRQ	0.004766	0.124341	1.775698	0	0.252902	0.042305	0.038454	0	0	0.024629	0	0	0	0.029650	0.015602	0	0	0.015289	0	0	0	0.014822	0.025996	0.020254	0.017076	0	0	0.021053	0	0	0	0.014060	39.72974	0.003692	not significant
CACDTVRTGGYAWDTRQ	0	0.006544	0	0.000461	0	0.001143	0	0	0	0.001296	0	0	0	0.001647	0	0.002191	0	0	0	0	0	0.002371	0	0	0	0.004899	0	0	0	0	0	0	0	5.396399	not significant

Supplementary Table 5. *STAT3/5B* mutations and immunophenotype of patients with γδ disease.

Pt: patient; HSTCL: Hepatosplenic T cell Lymphoma; LGLL: Large Granular Lymphocyte Leukemia; Y: yes; N: no; wt: wild type; Ly: Lymphocytes; n.d.: not determined. The symbols + and +/- indicate that the marker is present in the entire and in partial TCRγδ cell population, respectively; the symbol - indicates the absence of the marker.

Patients	Disease	Symptomatic disease	*STAT3* mutations	*STAT5B* mutations	Tγδ Ly (on Ly)	TCRγδ Lymphocytes																	
						Vδ1	Vδ2	Vγ9	CD5	CD8	CD16	CD56	CD57	CD158a	CD158b	CD158e	NKG2A	NKG2C	HLA-DR	CD28	CD45RA	CD45RO	CD62L
pt #1	HSTCL	Y	wt	V712E	94%	+	-	-	-	+	+	+	-	+/-	+	+	-	+	-	n.d.	+	n.d.	n.d.
pt #2	HSTCL	Y	wt	N642H	95%	+	-	-	-	-	+	+	-	+	+	-	-	+	-	n.d.	n.d.	n.d.	n.d.
pt #3	TCRγδ LGLL	Y	K658R, I659_M660insL	wt	74%	-	-	-	-	-	+	-	+	-	-	-	-	-	-	n.d.	n.d.	n.d.	n.d.
pt #4	TCRγδ LGLL	Y	D661Y	wt	71%	+	-	-	+/-	+	+	-	+	-	+	+	+	-	-	-	+	+	-
pt #5	TCRγδ LGLL	Y	wt	wt	50%	+	-	-	+	+/-	+	-	+	-	+	-	-	+	n.d.	n.d.	n.d.	n.d.	n.d.
pt #6	TCRγδ LGLL	Y	wt	wt	36%	+/-	-	-	+	+	+	-	+	-	+	-	-	+	-	-	+	+	-
pt #7	TCRγδ LGLL	N	wt	N642H, Q706L	64%	-	+	+	+	+	+	+	+	-	-	-	+	-	-	-	+	+	+
pt #8	TCRγδ LGLL	N	wt	N642H, L643M	44%	-	+	+	+	+	+	+	+	+	-	-	+	-	-	-	+	+	+
pt #9	TCRγδ LGLL	N	wt	Y665F	95%	-	+	+	+	+	+	+	+	-	-	-	+	-	n.d.	n.d.	n.d.	n.d.	n.d.
pt #10	TCRγδ LGLL	N	wt	wt	39%	-	+	+	+	+/-	+	+	+	-	-	-	-	-	-	-	+	+	+
pt #11	TCRγδ LGLL	N	wt	wt	30%	-	+	+	+	-	+	+	+	-	-	-	+	-	-	-	+	+	-
pt #12	HSTCL	Y	wt	Y665F	30%	-	-	-	-	-	+	+	-	+	+	+	-	+	-	-	-	+	-
pt #13	TCRγδ LGLL	N	wt	wt	19%	-	+	+	+	+	+	-	+	-	-	+	+	-	-	-	+	+	-
pt #14	TCRγδ LGLL	Y	wt	wt	27%	-	+	+/-	+	-	+	+	+	-	-	+/-	+	-	-	n.d.	n.d.	n.d.	n.d.
pt #15	TCRγδ LGLL	Y	wt	wt	35%	+	-	-	+	+	+	-	+/-	-	-	-	-	-	-	-	+	+	-
pt #16	TCRγδ LGLL	N	wt	wt	28%	-	+/-	+	+	+/-	+	+	-	-	-	-	+	-	-	-	+	+	-
pt #17	TCRγδ LGLL	N	wt	wt	20%	-	+	+	+	+/-	+	+	+	-	+/-	+	+	-	-	-	-	+	-
pt #18	TCRγδ LGLL	Y	wt	wt	25%	-	+	+	+	-	+	+	+	-	+	-	+	-	-	-	-	+	-
pt #19	TCRγδ LGLL	N	wt	wt	17%	-	+	+	+	-	+	+	+	+	+	-	-	-	-	-	+	+	-
pt #20	TCRγδ LGLL	N	wt	wt	21%	-	+	+	+	+	+	-	+	-	-	-	+	-	-	-	+	+	-
pt #21	TCRγδ LGLL	N	wt	wt	20%	-	+	+	+	-	+	-	+	-	+	-	+	-	-	n.d.	n.d.	n.d.	n.d.
pt #22	TCRγδ LGLL	N	wt	wt	18%	-	+	+/-	+	+	-	+	+	-	-	-	+	-	-	-	+	+	+
pt #23	TCRγδ LGLL	N	wt	wt	25%	-	+	+/-	+	+/-	+	-	+	+	+	-	-	-	n.d.	n.d.	n.d.	n.d.	n.d.
pt #24	TCRγδ LGLL	N	wt	wt	18%	-	+	+	+	-	+	+	+	-	-	-	+	-	-	-	+	+	-
pt #25	TCRγδ LGLL	Y	wt	wt	18%	+/-	-	-	-	-	-	-	+	-	-	-	-	-	-	n.d.	n.d.	n.d.	n.d.
pt #26	TCRγδ LGLL	Y	wt	wt	23%	+	-	-	+	+	+	-	+	-	-	-	-	-	+	n.d.	n.d.	n.d.	n.d.
pt #27	TCRγδ LGLL	N	wt	wt	28%	-	+	+	+	+/-	+	+	+	-	-	-	+	-	-	-	+	+	+
pt #28	TCRγδ LGLL	N	wt	wt	25%	-	+	+	+	+	+	+	+	-	-	-	-	-	-	-	+	-	+
pt #29	TCRγδ LGLL	Y	Y640F	wt	58%	-	-	-	-	+/-	+	-	+	-	-	-	-	-	-	-	+	-	-
pt #30	TCRγδ LGLL	N	wt	wt	27%	-	+	+	+	+	+	+	+	-	-	-	+	-	-	-	+	+	+
pt #31	TCRγδ LGLL	Y	wt	wt	25%	+	-	-	-	-	+	-	+	-	+	-	-	-	-	-	+	-	-
pt #32	TCRγδ LGLL	N	wt	wt	21%	-	+	+	+	-	-	+	-	-	-	-	+	-	-	n.d.	n.d.	n.d.	n.d.
pt #33	TCRγδ LGLL	Y	D661Y	wt	60%	+	-	-	+	+/-	+	-	+	-	-	-	-	-	-	-	+	-	-
pt #34	TCRγδ LGLL	Y	wt	wt	84%	+	-	-	-	-	+	+	-	-	-	-	-	+	-	-	+	+	-
pt #35	TCRγδ LGLL	Y	Y640F	wt	40%	-	-	+/-	-	+	+	-	+	-	-	-	-	-	-	-	+	-	-
pt #36	TCRγδ LGLL	Y	Y640F	wt	44%	+	-	-	-	+	+	-	+/-	n.d.	n.d.	n.d.	n.d.	n.d.	-	n.d.	n.d.	n.d.	n.d.
pt #37	TCRγδ LGLL	Y	D661Y	wt	77%	-	-	-	-	+/-	+	-	+	-	-	-	-	-	-	-	+	-	-
pt #38	TCRγδ LGLL	Y	wt	wt	16%	+	-	-	+	+	+	+	+	-	+	-	-	-	-	-	+	+	-
pt #39	TCRγδ LGLL	N	wt	wt	30%	-	+	+	-	+	+	+	+	-	+/-	-	-	-	+	-	+	+	-

Supplementary Table 6. *STAT3/5B* mutations and clinical features of patients with γδ disease.
Pt: patient; HSTCL: Hepatosplenic T cell Lymphoma; LGLL: Large Granular Lymphocyte Leukemia; M: male; F: female; Ly: Lymphocytes; n.d.: not determined; wt: wild type; Y: yes; N: no; Hb: hemoglobin; ANC: Absolute Neutrophils Count; PLT: Platelets; AIHA: autoimmune hemolytic anemia; RA: rheumatoid arthritis; SLE: systemic lupus erythematosus; ITP: immune thrombocytopenia; BS: Behçet Syndrome; APS: Autoimmune Polyendocrine Syndrome; RS: Raynaud Syndrome; CIN: Cervical Intraepithelial Neoplasia; MGUS: Monoclonal Gammopathy of Undetermined Significance. * indicates values under the physiologic range, indicating anemia, neutropenia and thrombocytopenia. "Symptomatic disease" is referred to patients with cytopenia and/or constitutional symptoms.

Patients	Disease	Tγδ Ly (on Ly)	Tγδ Ly (cells/µl)	*STAT3* mutations	*STAT5B* mutations	Symptomatic disease	Hb g/L	ANC 10^9/L	PLT 10^9/L	Autoimmune disease	Splenomegaly	Secondary Neoplasia	Therapy	Exitus
pt #1	HSTCL	94%	61670	wt	V712E	Y	90*	3.48	70*	N	Y	N	Y	deceased
pt #2	HSTCL	95%	7350	wt	N642H	Y	90*	4.05	22*	N	Y	N	Y	deceased
pt #3	TCRγδ LGLL	74%	1119	K658R, I659_M660ins	wt	Y	75*	0.96*	307	N	N	Thymoma	Y	alive
pt #4	TCRγδ LGLL	71%	3114	D661Y	wt	Y	70*	0.08*	162	AIHA, RA	Y	N	Y	deceased
pt #5	TCRγδ LGLL	50%	n.d.	wt	wt	Y	78*	2.10	89*	SLE	N	N	Y	alive
pt #6	TCRγδ LGLL	36%	1423	wt	wt	Y	123	0.40*	9*	ITP	Y	Thymoma	N	alive
pt #7	TCRγδ LGLL	64%	1391	wt	N642H, Q706L	N	132	2.00	276	N	N	N	N	alive
pt #8	TCRγδ LGLL	44%	1005	wt	N642H, L643M	N	150	2.46	205	N	N	N	N	alive
pt #9	TCRγδ LGLL	95%	4528	wt	Y665F	N	148	5.91	219	N	N	N	N	alive
pt #10	TCRγδ LGLL	39%	1306	wt	wt	N	154	1.84	304	N	N	N	N	alive
pt #11	TCRγδ LGLL	30%	1055	wt	wt	N	141	3.77	232	N	N	Basalioma	N	alive
pt #12	HSTCL	30%	111	wt	Y665F	Y	121	0.20*	65*	N	Y	N	Y	alive
pt #13	TCRγδ LGLL	19%	630	wt	wt	N	148	4.42	165	N	N	N	N	alive
pt #14	TCRγδ LGLL	27%	236	wt	wt	Y	114*	0.33*	147	N	N	N	N	alive
pt #15	TCRγδ LGLL	35%	319	wt	wt	Y	90*	0.62*	189	APS, AIHA	Y	N	Y	alive
pt #16	TCRγδ LGLL	28%	883	wt	wt	N	145	8.50	263	N	N	N	N	alive
pt #17	TCRγδ LGLL	20%	532	wt	wt	N	144	4.49	320	ITP	N	N	N	alive
pt #18	TCRγδ LGLL	25%	459	wt	wt	Y	128	1.20*	172	N	N	Follicular Adenoma	N	alive
pt #19	TCRγδ LGLL	17%	545	wt	wt	N	137	3.14	293	N	N	N	N	alive
pt #20	TCRγδ LGLL	21%	283	wt	wt	N	145	2.90	212	N	N	N	N	alive
pt #21	TCRγδ LGLL	20%	767	wt	wt	N	140	3.64	246	N	N	N	N	alive
pt #22	TCRγδ LGLL	18%	264	wt	wt	N	157	2.37	228	N	N	N	N	alive
pt #23	TCRγδ LGLL	25%	724	wt	wt	N	147	3.70	268	N	N	N	N	alive
pt #24	TCRγδ LGLL	18%	792	wt	wt	N	132	3.52	319	N	N	N	N	alive
pt #25	TCRγδ LGLL	18%	502	wt	wt	Y	121	0.20*	271	N	N	N	Y	alive
pt #26	TCRγδ LGLL	23%	2557	wt	wt	Y	112*	1.40*	304	N	N	N	N	alive
pt #27	TCRγδ LGLL	28%	648	wt	wt	N	155	2.30	255	BS	N	Seminoma	N	alive
pt #28	TCRγδ LGLL	25%	292	wt	wt	N	142	8.04	238	N	N	N	N	alive
pt #29	TCRγδ LGLL	58%	1480	Y640F	wt	Y	95*	0.52*	118	Vitiligo, RS, AIHA	Y	N	Y	alive
pt #30	TCRγδ LGLL	27%	476	wt	wt	N	144	2.30	194	N	N	N	N	alive
pt #31	TCRγδ LGLL	25%	1088	wt	wt	Y	149	0.97*	215	N	N	N	N	alive
pt #32	TCRγδ LGLL	21%	503	wt	wt	N	122	4.35	386	N	N	N	N	alive
pt #33	TCRγδ LGLL	60%	3994	D661Y	wt	Y	136	1.33*	341	N	N	N	N	alive
pt #34	TCRγδ LGLL	84%	5820	wt	wt	Y	76*	0.93*	537	N	N	N	Y	alive
pt #35	TCRγδ LGLL	40%	1147	Y640F	wt	Y	127	1.26*	230	N	N	N	N	alive
pt #36	TCRγδ LGLL	44%	105	Y640F	wt	Y	130	0.11*	152	N	N	CIN	N	alive
pt #37	TCRγδ LGLL	77%	4421	D661Y	wt	Y	111*	1.44*	272	RA	N	Tyroid Adenoma	N	alive
pt #38	TCRγδ LGLL	16%	199	wt	wt	Y	139	0.73*	150	N	N	N	N	alive
pt #39	TCRγδ LGLL	30%	725	wt	wt	N	131	2.16	216	N	N	MGUS	N	alive
