# Supplementary material for: Defining TCRγδ lymphoproliferative disorders by combined immunophenotypic and molecular evaluation
Source: Nat Commun. 2022 Jun 8;13:3298. doi: 10.1038/s41467-022-31015-x (PMC9177852; doi:10.1038/s41467-022-31015-x)
Supplement: Supplementary file 2 — Reporting Summary [file 41467_2022_31015_MOESM2_ESM.pdf]

nature portfolio

Corresponding author(s): Prof. Renato Zambello, Prof. Gianpietro Semenzato

Last updated by author(s): May 23, 2022

Reporting Summary

Nature Portfolio wishes to improve the reproducibility of the work that we publish. This form provides structure for consistency and transparency in reporting. For further information on Nature Portfolio policies, see our Editorial Policies and the Editorial Policy Checklist.

Statistics

For all statistical analyses, confirm that the following items are present in the figure legend, table legend, main text, or Methods section.

n/a	Confirmed	
☐	☒	The exact sample size (*n*) for each experimental group/condition, given as a discrete number and unit of measurement
☐	☒	A statement on whether measurements were taken from distinct samples or whether the same sample was measured repeatedly
☐	☒	The statistical test(s) used AND whether they are one- or two-sided<br>*Only common tests should be described solely by name; describe more complex techniques in the Methods section.*
☒	☐	A description of all covariates tested
☐	☒	A description of any assumptions or corrections, such as tests of normality and adjustment for multiple comparisons
☐	☒	A full description of the statistical parameters including central tendency (e.g. means) or other basic estimates (e.g. regression coefficient) AND variation (e.g. standard deviation) or associated estimates of uncertainty (e.g. confidence intervals)
☐	☒	For null hypothesis testing, the test statistic (e.g. *F*, *t*, *r*) with confidence intervals, effect sizes, degrees of freedom and *P* value noted<br>*Give P values as exact values whenever suitable.*
☒	☐	For Bayesian analysis, information on the choice of priors and Markov chain Monte Carlo settings
☐	☒	For hierarchical and complex designs, identification of the appropriate level for tests and full reporting of outcomes
☒	☐	Estimates of effect sizes (e.g. Cohen's *d*, Pearson's *r*), indicating how they were calculated

Our web collection on statistics for biologists contains articles on many of the points above.

Software and code

Policy information about availability of computer code

Data collection	For TCR gamma chain sequencing: LymphoTrack Dx TRG Panel-MiSeq. Ref. 9-227-0009; for TCR delta chain sequencing: Euroclonality-NGS working group protocol (www.euroclonality.org/protocols/); for STAT3 and STAT5b amplicon sequencing: TruSeq Custom Amplicon Assay 1.5 (Illumina); for cytometer analysis: FACSCanto analyzer II.
Data analysis	LymphoTrackDxMiseq2.4.3, MiXCR v3.0.13, VDJtools v1.2.1, ClustalW web tool (https://www.genome.jp/tools-bin/clustalw), WebLogo web tool (https://weblogo.berkeley.edu/logo.cgi), Illumina VariantStudio software v2.2, FACSDiva software v8.0.2, GraphPad Prism 6 software, Chromas Pro v1.5 and Blast (https://blast.ncbi.nlm.nih.gov/Blast.cgi).

For manuscripts utilizing custom algorithms or software that are central to the research but not yet described in published literature, software must be made available to editors and reviewers. We strongly encourage code deposition in a community repository (e.g. GitHub). See the Nature Portfolio guidelines for submitting code & software for further information.

Data

Policy information about availability of data

All manuscripts must include a data availability statement. This statement should provide the following information, where applicable:

- Accession codes, unique identifiers, or web links for publicly available datasets
- A description of any restrictions on data availability
- For clinical datasets or third party data, please ensure that the statement adheres to our policy

TCR NGS and STAT gene NGS data are included in a BioProject in NCBI SRA database (PRJNA715076), publicly available [https://www.ncbi.nlm.nih.gov/bioproject/PRJNA715076].

Field-specific reporting

Please select the one below that is the best fit for your research. If you are not sure, read the appropriate sections before making your selection.

☒ Life sciences ☐ Behavioural & social sciences ☐ Ecological, evolutionary & environmental sciences

For a reference copy of the document with all sections, see nature.com/documents/nr-reporting-summary-flat.pdf

Life sciences study design

All studies must disclose on these points even when the disclosure is negative.

Sample size	The entire cohort of the study included a total of 39 patients (36 Tγδ large granular lymphocyte leukemia, LGLL, and 3 hepatosplenic T cell lymphoma, HSTCL), comprehensive of a main group of 9 Tγδ LGLL and 2 HSTCL, analyzed by NGS for clonotype repertoire and STAT mutations, and a larger retrospective cohort including 28 patients for which we had already collected immunophenotype data, genetic status of STAT3/STAT5B mutations by Sanger sequencing and clinical course. The patients affected by HSTCL expressing TCRγδ were included in the study for comparison. The number of cases studied was not determined by sample-size calculation, but accordingly to the availability of good-quality DNA samples for NGS, since TCRγδ LGLL and HSTCL were very rare neoplasia and the number of collected samples is the result of years samples' collection in Padova Hematology Unit, reference center for LGLL diseases.<br>The 23 healthy donors were collected to have at least the double number of the patients.
Data exclusions	No data were excluded for the analyses.
Replication	The immunophenotype data obtained by cytometer have been replicated along more time points and the results reported in the paper are all confirmed by at least 2 evaluations. All the mutations found by amplicon sequencing were confirmed by Sanger sequencing. Two samples for TCR gamma chain and all the samples for TCR delta chain were repeated twice by NGS and the results were all confirmed (data not reported in the paper but available upon request).
Randomization	The patients were recruited according to 2017 WHO diagnostic criteria. The controls were healthy adult volunteers. Samples in our study were not randomized into experimental groups. The allocation in the experiments was based on the availability and quality of the collected samples.
Blinding	Different investigators collected immunophenotype results, Sanger sequencing data, NGS data and bioinformatic analysis, and all were blinded on the clinical features of the study cases (patients/healthy/symptomatic/indolent disease).

Reporting for specific materials, systems and methods

We require information from authors about some types of materials, experimental systems and methods used in many studies. Here, indicate whether each material, system or method listed is relevant to your study. If you are not sure if a list item applies to your research, read the appropriate section before selecting a response.

Materials & experimental systems

n/a	Involved in the study	
☐	☒	Antibodies
☒	☐	Eukaryotic cell lines
☒	☐	Palaeontology and archaeology
☒	☐	Animals and other organisms
☐	☒	Human research participants
☒	☐	Clinical data
☒	☐	Dual use research of concern

Methods

n/a	Involved in the study	
☒	☐	ChIP-seq
☐	☒	Flow cytometry
☒	☐	MRI-based neuroimaging

Antibodies

Antibodies used	Antibodies from Becton Dickinson: CD3 APC (SK7, cod. 345767), CD3 PE-CY7 (SK7, cod. 341111), CD3 APC-CY7 (SK7, cod. 341110), CD4 FITC (SK3, cod. 345768) , CD5 PE-CY7 (L17F12, cod. 348810), CD8 PE (SK1, cod. 345773), CD16 FITC (NKP15, cod. 335035), CD16 PERCP-CY5.5 (3G8, cod. 338440), CD28 PE (L293, cod. 348047), CD45RA PE-CY7 (L48, cod. 337186), CD45RO APC (UCHL1, cod. 340438), CD56 PE-CY7 (NCAM16.2, cod. 335826), CD56 APC (NCAM16.2, cod. 341027), CD57 FITC (333169, cod. 333169), CD62L PERCP (DREG-56, cod. 555545), CD94 FITC (HP-3D9, cod. 555888), HLA-DR PERCP (L243, cod. 347402), TCRγδ FITC (11F2, cod. 347903), TCRγδ PE (11F2, cod. 333141), CD158a FITC (HP-3E4, cod. 340531), CD158b PE (CH-L, cod. 559785), CD158e PE (DX9, cod. 340484). Antibodies from R&D Systems: NKG2A PE (131411, cod. FAB1059P) and NKG2C APC (134591, cod. FAB138A). Antibodies from Thermo Fisher Scientific: Vγ9 FITC (7A5, cod. TCR2720), Vδ1 FITC (TS8.2, cod. TCR2730) and Vδ2 FITC (15D, cod. TCR2732).
Validation	All antibodies were validated by the manufacturer.

Human research participants

Policy information about studies involving human research participants

Population characteristics	The relevant characteristics of the patients recruited in this study included the clonality of Tgd lymphocytes in peripheral blood (in Tgd-LGLL persisting more than 6 months), adult age (range: 35-92), no gender specificity, no cromosomal abnormalities. The clinical data are reported in Supplementary Table 6. All the samples evaluated in this study were obtained from patients out of or pre-therapy. For clonotype repertoire evaluation by NGS performed in the pilot group, a control group of 23 samples represented by healthy blood donors was included. The control group was characterized by age (median: 64, IQR: 51-70) and gender (M/F: 12/11) matching the values of the patients' group (median age: 65, IQR: 55-74; M/F: 5/6).
Recruitment	All patients were recruited by the Hematology Unit of Padova Hospital since 2001 and retrospectively revised to meet 2017 WHO criteria for T-LGLL or HSTCL diagnosis. Patients were recruited by healthcare professionals independent from the study investigators, and samples were obtained without bias according to samples' availability. Controls were healthy adult volunteer participants characterized by age and gender matching the values of the patients' group.
Ethics oversight	This study was performed according to the Helsinki Declaration and patients gave written informed consensus before their inclusion in the study. The study and blood sample collection were approved by the Padova University Hospital Ethics Committee (approval number 4213/AO/17).

Note that full information on the approval of the study protocol must also be provided in the manuscript.

Flow Cytometry

Plots

Confirm that:

☒ The axis labels state the marker and fluorochrome used (e.g. CD4-FITC).

☒ The axis scales are clearly visible. Include numbers along axes only for bottom left plot of group (a 'group' is an analysis of identical markers).

☐ All plots are contour plots with outliers or pseudocolor plots.

☐ A numerical value for number of cells or percentage (with statistics) is provided.

Methodology

Sample preparation	PBMC purified from peripheral blood human samples
Instrument	FACSCanto analyzer II
Software	FACSDiva software v8.0.2 Cell population
Cell population abundance	The flow cytometric evaluation documented an increase of Tγδ cell percentage (16%-94% of blood lymphocytes) in all the patients.
Gating strategy	The flow analysis is performed on peripheral blood mononuclear cells (PBMC). FSC-H vs FSC-A plot allowed to exclude doublets. Then, in the SSC-A vs FSC-A plot a morphologic gate was designed to identify lymphocytes within PBMC. PBMC were stained with CD3-APC, TCRγδ-PE, Vδ1-FITC, Vδ2-FITC and Vγ9-FITC to identify and characterize the percentage of Tγδ cells on lymphocytes. CD3+/Tγδ cell markers (CD5, CD8, HLA-DR, CD16, CD28, CD45RA, CD45RO, CD56, CD57, CD62L, CD94, CD158a, CD158b, CD158e, NKG2A, NKG2C) were determined by staining cells using two different anti-TCRγδ antibodies, i.e. TCRγδ FITC and TCRγδ PE, according to the multicolor flow cytometry panel design. The gating strategy is provided as Supplementary Figure 5.

☒ Tick this box to confirm that a figure exemplifying the gating strategy is provided in the Supplementary Information.
